# Constraint-Based Modeling Highlights Cell Energy, Redox Status and α-Ketoglutarate Availability as Metabolic Drivers for Anthocyanin Accumulation in Grape Cells Under Nitrogen Limitation

**DOI:** 10.3389/fpls.2018.00421

**Published:** 2018-05-17

**Authors:** Eric Soubeyrand, Sophie Colombié, Bertrand Beauvoit, Zhanwu Dai, Stéphanie Cluzet, Ghislaine Hilbert, Christel Renaud, Lilly Maneta-Peyret, Martine Dieuaide-Noubhani, Jean-Michel Mérillon, Yves Gibon, Serge Delrot, Eric Gomès

**Affiliations:** ^1^UMR 1287 Ecophysiologie et Génomique Fonctionnelle de la Vigne, Université de Bordeaux, Institut des Sciences de la Vigne et du Vin, Bordeaux, France; ^2^UMR 1332 Biologie du Fruit et Pathologie, INRA-Bordeaux, IBVM, Bordeaux, France; ^3^UMR 1287 Ecophysiologie et Génomique Fonctionnelle de la Vigne, INRA-Bordeaux, Institut des Sciences de la Vigne et du Vin, Bordeaux, France; ^4^EA 3675 GESVAB, Université de Bordeaux, Institut des Sciences de la Vigne et du Vin, Bordeaux, France; ^5^UMR 5200 Laboratoire de Biogenèse Membranaire, Université de Bordeaux, Bordeaux, France; ^6^UMR 1332 Biologie du Fruit et Pathologie, Université de Bordeaux, IBVM, Bordeaux, France

**Keywords:** anthocyanins, grapevine, cell redox status, energy escape valve hypothesis, constraint-based modeling

## Abstract

Anthocyanin biosynthesis is regulated by environmental factors (such as light, temperature, and water availability) and nutrient status (such as carbon, nitrogen, and phosphate nutrition). Previous reports show that low nitrogen availability strongly enhances anthocyanin accumulation in non carbon-limited plant organs or cell suspensions. It has been hypothesized that high carbon-to-nitrogen ratio would lead to an energy excess in plant cells, and that an increase in flavonoid pathway metabolic fluxes would act as an “energy escape valve,” helping plant cells to cope with energy and carbon excess. However, this hypothesis has never been tested directly. To this end, we used the grapevine *Vitis vinifera* L. cultivar Gamay Teinturier (syn. Gamay Freaux or Freaux Tintorier, VIVC #4382) cell suspension line as a model system to study the regulation of anthocyanin accumulation in response to nitrogen supply. The cells were sub-cultured in the presence of either control (25 mM) or low (5 mM) nitrate concentration. Targeted metabolomics and enzyme activity determinations were used to parametrize a constraint-based model describing both the central carbon and nitrogen metabolisms and the flavonoid (phenylpropanoid) pathway connected by the energy (ATP) and reducing power equivalents (NADPH and NADH) cofactors. The flux analysis (2 flux maps generated, for control and low nitrogen in culture medium) clearly showed that in low nitrogen-fed cells all the metabolic fluxes of central metabolism were decreased, whereas fluxes that consume energy and reducing power, were either increased (upper part of glycolysis, shikimate, and flavonoid pathway) or maintained (pentose phosphate pathway). Also, fluxes of flavanone 3β-hydroxylase, flavonol synthase, and anthocyanidin synthase were strongly increased, advocating for a regulation of the flavonoid pathway by alpha-ketoglutarate levels. These results strongly support the hypothesis of anthocyanin biosynthesis acting as an energy escape valve in plant cells, and they open new possibilities to manipulate flavonoid production in plant cells. They do not, however, support a role of anthocyanins as an effective mechanism for coping with carbon excess in high carbon to nitrogen ratio situations in grape cells. Instead, constraint-based modeling output and biomass analysis indicate that carbon excess is dealt with by vacuolar storage of soluble sugars.

## Introduction

Flavonoids are naturally occurring secondary metabolites belonging to the group of polyphenols, which are ubiquitous in all land plants, with currently over 9,000 compounds identified (Buer et al., 2010). Among polyphenols, flavonoids encompass over 6000 distinct molecules, divided into aurones, flavones, flavonols, flavanols, anthocyanins, phlobaphenes, and isoflavonoids, the last two being almost exclusively synthesized in maize and leguminous plants (Hichri et al., 2011). They exhibit a large variety of biological roles in plants. They control pollen fertility in many species (Taylor and Jorgensen, 1992) and influence auxin transport (Peer and Murphy, 2007). Light absorbing pigments such as anthocyanins and aurones color flower petals and fruit epicarp, thus facilitating pollinator attraction and seed dispersal (Mol et al., 1998). With regard to human health, the consumption of grapes or grape-derived products, has been correlated with a reduced incidence of a number of chronic illnesses (Iriti and Faoro, 2009; Kozlowska and Szostak-Wegierek, 2014), and flavonoids have been proposed as major contributors of these health-promoting effects (Butelli et al., 2008; Qin et al., 2011).

**FIGURE 1:**
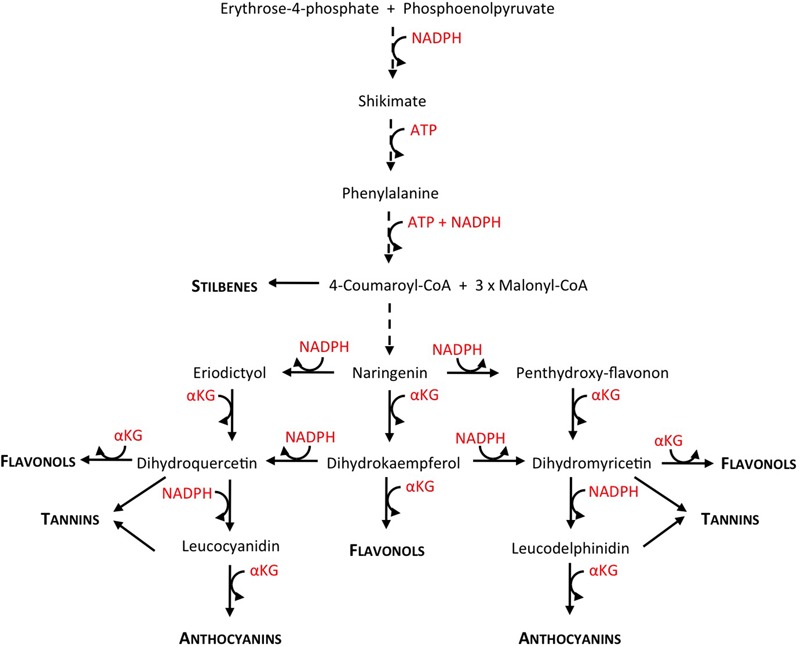
Comprehensive view of the polyphenol biosynthetic pathway, showing NADPH, ATP, and αKG consumption (reconstructed from KEGG pathway database http://www.genome.jp/kegg/pathway.html). Dashed line arrows indicate more than one enzymatic step. Biosynthetic intermediates are written in regular letters, end products in small capitals and bold.

Anthocyanins, which are key compounds for premium red wine making, are present in the skin (epicarp) of the red grape berries, and sometimes, in the case of the so-called “teinturier” cultivars also in the pulp (mesocarp) (Petrussa et al., 2011; Guan et al., 2016). Hence, in order to optimize anthocyanin content in the berries, it is important to understand the molecular regulation of the anthocyanin production by environmental factors and viticultural practices. Anthocyanins are synthesized through the phenylpropanoid and flavonoid pathways, starting with phenylalanine as a precursor, and splitting into two branches to produce the di- and tri-hydroxylated flavonoids (**Figure 1**; Tanaka et al., 2008; He et al., 2010). The accumulation and the proportion of these compounds in the berry skin depends on genetic, developmental, and environmental factors (Rio Senegade et al., 2008; He et al., 2010; Dai et al., 2011) as well as on viticultural practices (Downey et al., 2006). Light, temperature, irrigation, and nitrogen supply have been shown to impact grape berry anthocyanin content (Dai et al., 2011; Berdeja et al., 2015; Keller, 2015; Habran et al., 2016).

In several crops like tomato or grapevine and model plants such as Arabidopsis, nitrogen depletion increases the concentration of phenolics in general and of anthocyanins in particular (Keller and Hrazdina, 1998; Hilbert et al., 2003; Fritz et al., 2006; Feyissa et al., 2009; Løvdal et al., 2010; Soubeyrand et al., 2014; Habran et al., 2016). The transcriptional regulation of the phenylpropanoid and flavonoid pathways in response to low nitrogen availability has been extensively studied in Arabidopsis (Lillo et al., 2008), tomato (Løvdal et al., 2010), tobacco (Fritz et al., 2006), or grapevine (Soubeyrand et al., 2014). These studies shed light on the molecular mechanisms underlying the regulation of the flavonoid metabolism by nitrogen depletion, pointing out the responsiveness of the pathway’s positive and negative regulators, i.e., R2-R3MYB and LBD transcription factors. They do not, however, address the pending question of the existence of a metabolic “driver” that would fuel the increase in flavonoid biosynthesis. According to Hernández and Van Breusegem (2010), a plausible candidate for such a metabolic driver could be the cell energetic status. High carbon-to-nitrogen ratio leads to an energy excess, both in terms of high ATP and high reducing power (NADH and NADPH); and an increase in flavonoid biosynthetic metabolic fluxes would act as an “energy escape valve,” helping plant cells to cope with that energy excess. Indeed, the flavonoid pathway consumes ATP and NADPH reducing equivalents in several of its enzymatic steps particularly when the shikimate pathway, that links the central metabolism to the phenylpropanoid pathway, is also taken into account (**Figure 1**).

Constraint-based modeling can be used in order to test that hypothesis by comparing maps of metabolic fluxes in the two contrasted situations, i.e., in nitrogen limiting condition compared to the control condition. Mathematical modeling of metabolism is a particularly promising tool as it offers a systems approach to analyze the structure, dynamics, and behavior of complex metabolic networks. In plant research, the issue of modeling metabolism is increasingly gaining attention, and several mathematical modeling approaches applied to plant metabolism exist (for reviews, see Giersch, 2000; Morgan and Rhodes, 2002; Poolman et al., 2004; Rios-Estepa and Lange, 2007). Constraint-based modeling such as flux balance analysis (FBA, Orth et al., 2010) allows the prediction of metabolic fluxes at steady-state by applying mass balance constraints to a stoichiometric model. Mass-balance information, such as growth rate, biomass composition, and substrate consumption rate, are used to fix boundaries on the flux solutions space (Reed and Palsson, 2003) and an objective function is used to identify the optimal flux distribution among all possible steady-state flux distributions. This modeling has the advantage of not requiring the knowledge of enzyme kinetic parameters.

The present work aims to investigate the metabolic flux reorganization that is involved in the response of anthocyanin accumulation to nitrogen supply, taking advantage of a grapevine red cell suspension system, the GT3 *Vitis vinifera* L., cv. Gamay Teinturier (syn. Freaux or Gamay Freaux Tintorier, Vitis International Variety Catalogue #4382, Decendit and Mérillon, 1996). To this end, the cells were cultivated in control or nitrogen limiting conditions. Then we generated and compared flux maps of plant cell metabolism by coupling the network of heterotrophic metabolism previously described (Colombié et al., 2015) with the overall reactions to phenolic compounds production (anthocyanins, flavonols, tannins, and stilbenes), paying special attention to energetic processes by balancing cofactors. The results are consistent with excessive ATP and reducing equivalent (NADPH mostly) as well as α-ketoglutarate availability acting as “pushers” that increase anthocyanin and more broadly polyphenol biosynthesis in nitrogen-depleted cells.

## Materials and Methods

### Grapevine Cell Culture Growth and Sampling

*Vitis vinifera* cv. “Gamay Fréaux” var. teinturier GT3 cell suspensions were sub-cultured on a modified Gamborg B5 medium, supplemented with 20 g L^-1^ sucrose, 250 mg L^-1^ casein hydrolysate, 0.1 mg mL^-1^ 1-naphthalene acetic acid and 0.2 mg mL^-1^ kinetin (Saigne-Soulard et al., 2006). Cells were routinely sub-cultured every 7 days in 250 mL Erlenmeyer flasks containing 50 mL of culture medium. For experimental purpose, 7-days old cells were inoculated, with a 1:6 (v/v) ratio in 200 mL of the same medium but containing either 5 mM (final concentration, low nitrogen, N^-^) or 25 mM (final concentration, control, N) KNO_3_, in 1 L Erlenmeyer flasks. The ammonium concentration was identical in both N^-^ and N treatments (2 mM). For each sampling point, three replicate flasks of cell culture were harvested at 0 (or 1 for experiment 1), 4, 6, 8, and 11 days post-inoculation by vacuum filtration, quickly washed twice with ice-cold distillated water, weighed and quick-frozen in liquid nitrogen. Frozen cells were then reduced to fine powder in a liquid nitrogen-cooled MM200 ball grinder (Retsch, Haan, Germany), and stored at -80°C until further analysis.

### Cell Biomass and Metabolites Content Analysis

#### Phenolic Compounds

Anthocyanins and flavonols were analyzed on powdered freeze-dried cells, which were extracted and analyzed according to Soubeyrand et al. (2014).

Tannins and stilbenes were extracted from 40 mg of freeze-dried cells with 4 mL of methanol (100%) overnight at +4°C. The samples were centrifuged at 6,000 × *g* for 10 min. Two milliliters of supernatant were vacuum-dried using a SpeedVac SC 110 plus (Thermo Fisher Scientific, Saint-Herblain, France) for the analysis of stilbenes and 50 μL were used for the analysis of the total phenolic content. Then, 100 μL MeOH (100%) and 1 mL H_2_O were added to the vacuum-dried samples and filtered through an Ion Exchange Resin (Dowex 50 WX 4-400) to remove anthocyanins. Extracts were vacuum-dried using a SpeedVac SC 110 plus and the dry pellet was re-suspended in 800 μL of MeOH/H_2_0 50/50 (v/v) for the HPLC analysis. Extracts were then filtered through a 0.45 μm polypropylene syringe filter (Pall Gelman Sciences Corp., Ann Arbor, MI, United States). Stilbenes analysis was performed with a Summit HPLC System consisting of P680 pump, ASI-100T^TM^ autosampler and UVD 340U UV-Vis detector operating at 320 nm (Dionex Corporation, Sunnyvale, CA, United States) (Ramirez-Lopez and DeWitt, 2014). After injecting 20 μL, separation was achieved at ambient temperature on a reverse-phase Ultrasphere ODS column 25 cm × 4.6 mm, 5 μm particle size with an Ultrasphere ODS guard column 4.5 cm × 4.6 mm (Beckman Instruments Inc., Fullerton, CA, United States). All reagents were of analytical grade. Separation was performed according to Saigne-Soulard et al. (2006).

Total phenolic content was assessed by the Folin–Ciocalteu method (Singleton and Rossi, 1965). The assay mixture (3 mL) contained 50 μL of extract, 450 μL MeOH/H_2_O (50/50, v/v), 250 μL Folin–Ciocalteu reagent and 2.25 mL of ultrapure water. After 3 min, 2 mL Na_2_CO_3_ (75g L^-1^) were added and the samples were incubated at 50°C for 5 min and absorbance was read at 760 nm. Calculation of phenolics was based on a standard curve prepared using gallic acid, and the results were expressed as mg gallic acid equivalents per liter (mg GAE L^-1^).

#### Sugars and Amino Acids

Five hundred milligrams of cell powder (FW) were extracted from cell suspension samples using decreasing concentrations of ethanol: ethanol 80%, ethanol 50% (v/v) and ultrapure water. All three supernatants were pooled, vacuum-dried using a Speed Vac SC 110 plus (Thermo Fisher Scientific, Saint-Herblain, France). The dry pellet was re-suspended in 2 mL of ultrapure water and stored at -20°C before further analysis.

Amino-acid content was analyzed by the method described by Cohen and Michaud (1993), modified according to Martínez-Lüscher et al. (2014). Briefly, after derivatization with 6-aminoquinolyl-N-hydroxysuccinimidyl-carbamate, amino acids were analyzed using a Waters 2695 HPLC system equipped with a Waters 474 fluorescence detector (Waters, Milford, MA, United States). Separation was performed on a Nova-Pack C18 AccQ-Tag column (Waters, Milford, MA, United States) at 37°C with elution at 1 mL min^-1^ with a 67 min linear gradient (eluent A, sodium acetate buffer, 140 mM at pH 5.7; eluent B, acetonitrile 60% in water (v/v)). Chromatograms corresponding to excitation at 250 nm and emission at 395 nm were recorded and quantified with chemical standards purchased from Sigma (St. Louis, MO, United States).

Soluble sugars (glucose, fructose, and sucrose) were measured enzymatically with a microplate reader (ELx800UV, BioTek Instruments Inc., Winooski, VT, United States) as described by Gomez et al. (2007).

#### Malic Acid, Total Starch, and Proteins

Malate, starch, and protein content were measured as described in Biais et al. (2014).

#### Cell Wall Total Polysaccharides

Total cell wall polysaccharides quantification was subcontracted to the BIBS platform of INRA-Nantes^1^, using 100 mg of freeze-dried cell powder, as described in Colombié et al. (2015).

#### Lipids

Five hundred milligrams of cell powder (FW) were extracted by 1 mL of MeOH:H_2_SO_4_ (40:1, v/v), supplemented with 2 μg of heptadecanoic acid (internal standard) and incubated 60 min at 80°C in screw-capped tubes. Then, 400 μL hexane and 1.5 mL of ultrapure water were added, vigorously mixed and centrifuged at 3,000 *g* for 5 min. The organic phase was collected and transferred to injection vial to analyze fatty acids by GC-FID (Gas Chromatography coupled to Flame Ionization Detection), as described by Maneta-Peyret et al. (2014).

#### Total Carbon and Nitrogen Content

Cell total carbon and nitrogen contents were determined by Dumas’ combustion method, with a Flash EA 112 auto-analyzer (Thermo fisher, Courtaboeuf, France), following the manufacturer’s instructions and using 8 mg of freeze-dried cell powder. In the case of culture medium analysis, 250 μL of freeze-dried medium were used instead.

#### Total Nucleic Acid

The total DNA content was measured using the deoxyribose-specific diphenylamine reaction, using 15 mg of freeze-dried cell powder as starting material, and salmon sperm DNA for calibration (Colombié et al., 2015).

### Enzyme Capacity Determinations

Phenylalanine ammonia-lyase (PAL) activity was measured according to Gagné (2007). Approximately 250 mg of cell powder were extracted by vigorous shaking with 40 mg polyvinylpolypyrrolidone (PVPP) and 2.5 mL extraction buffer composed of 0.1 mM Tris–HCl (pH 8.8), 5 mM EDTA, 0.05% spermidine (w/v), 4 mM β-mercaptoethanol, and 1 mM phenylmethylsulfonyl fluoride (PMSF, added just prior extraction). The samples were centrifuged for 20 min at 16,000 × *g* at 4°C. The protein extract was desalted on a PD-10 column (Sephadex resin G-25, PD-10 column, GE Healthcare) equilibrated with 25 mL of 0.1 mM Tris–HCl (pH 8.8). Aliquots of desalted proteins were frozen in liquid nitrogen and stored at -80°C. Spectrophotometric assays contained 300 μL of protein extract in 30 mM L-phenylalanine in 0.1 M Tris–HCl (pH 8.8) and 150 μL of 30 mM L-phenylalanine in 0.1 M Tris–HCl (pH 8.8). Reactions were incubated for 15 to 180 min at 37°C. The amount of *trans*-cinnamic acid formed in the assay was measured spectrophotometrically at 290 nm. PAL activity was expressed as μg of cinnamic acid formed per μg FW^-1^.

For all other enzyme measurements [glucose-6-phosphate dehydrogenase (G6PDH), phosphoglucomutase (PGM), fructose hexokinase (FK), glucose hexokinase (GK), shikimate dehydrogenase (SD), enolase (ENO), pyruvate kinase (PK) and glutamine synthetase (GS)], aliquots of 20 mg frozen FW cell powder were extracted by vigorous mixing with extraction buffer (Nunes-Nesi et al., 2007). FK, GK, G6PDH, PK, SD, and GS were assayed as described in Gibon et al. (2004). ENO was assayed as described by Biais et al. (2014), PGM was assayed according to Gibon et al. (2009).

### Respiration Measurements

Oxygen consumption rates of cells were measured with Clark’s electrode at 25°C in a 1 mL thermostatically controlled chamber. Respiration assays of growing cells were performed in the GT3 cell suspension medium under stirring. Seven hundred and fifty microliters of cell suspension were centrifuged (1,500 × *g* for 5 min) and the resulting pellet gently re-suspended in 1 mL of cell culture medium. Respiration rates were initially expressed in nmol O_2_ min^-1^ g^-1^ FW.

### Coenzyme Analysis

All extractions were performed at 4°C with 200 mg of frozen powder cell. For the assays of NAD^+^ and NADP^+^, aliquots of frozen cells were extracted with 500 μL of 0.2 N HCl then incubated for 5 min at 80°C. Fifty microliters of 0.2 M NaH_2_PO_4_ (pH 5.6) was added and the extract was neutralized to a final pH in the range from 5.5 to 6.5 with 0.2 M NaOH. To quantify NADH and NADPH, other aliquots of frozen cells were extracted as for NAD^+^ and NADP^+^ except that the extraction medium was 0.2 M NaOH and the heated sample was neutralized with 0.2 N HCl to a final pH in the range from 7.5 to 8.5.

Coenzyme content was quantified by adapting methods described by Wilhelm (2009). The reaction buffer was composed by Tris/KOH (pH 7.7) qsp 350 μL, 100 μL of 10 mM methylthiazolyldiphenyl-tetrazolium (MTT) and 50 μL of 4 mM phenazine ethosulfate (PES). For the NAD^+^ and NADH assay, the reaction was started by adding 3.5 U of alcohol dehydrogenase (ADH, Roche, Melan, France) and 10 μL ethanol (99%). For the NADP^+^ and NADPH assay, 1.6 U of glucose-6-phosphate dehydrogenase (Roche) and 0.5 M glucose-6-phosphate were added to the assay. Absorbance was read at 570 nm for 10 min, and the results were expressed in nmol g^-1^ FW.

### Modeling

Concentrations of accumulated metabolites and biomass components were converted from gram-to mole-basis and then multiplied by the specific growth rate calculated at day 4 and 6 in order to calculate the corresponding fluxes used as constraints in the flux balance model. Stoichiometric network reconstruction encompassing central and polyphenol metabolism (model in *sbml* format, Supplementary Presentation 2) and mathematical problems were implemented using MATLAB (Mathworks R2012b, Natick, MA, United States) and the optimization toolbox, solver quadprod with interior-point-convex algorithm for the minimization.

### Statistical Analysis

Statistical analyses were done using the statistical package of the “R” software (R Development Core Team, 2010). A one-way analysis of variance (ANOVA) was used. Unless otherwise stated, the mean of the 3 biological replicate treatments was used in data analysis. Unless otherwise stated, comparisons of means were performed using HSD.r multiple comparisons function of Tukey’s *post hoc* test at *P* < 0.05.

## Results

### Anthocyanin Accumulation in Cells Cultivated Under Control and Low Nitrogen Conditions

**FIGURE 2:**
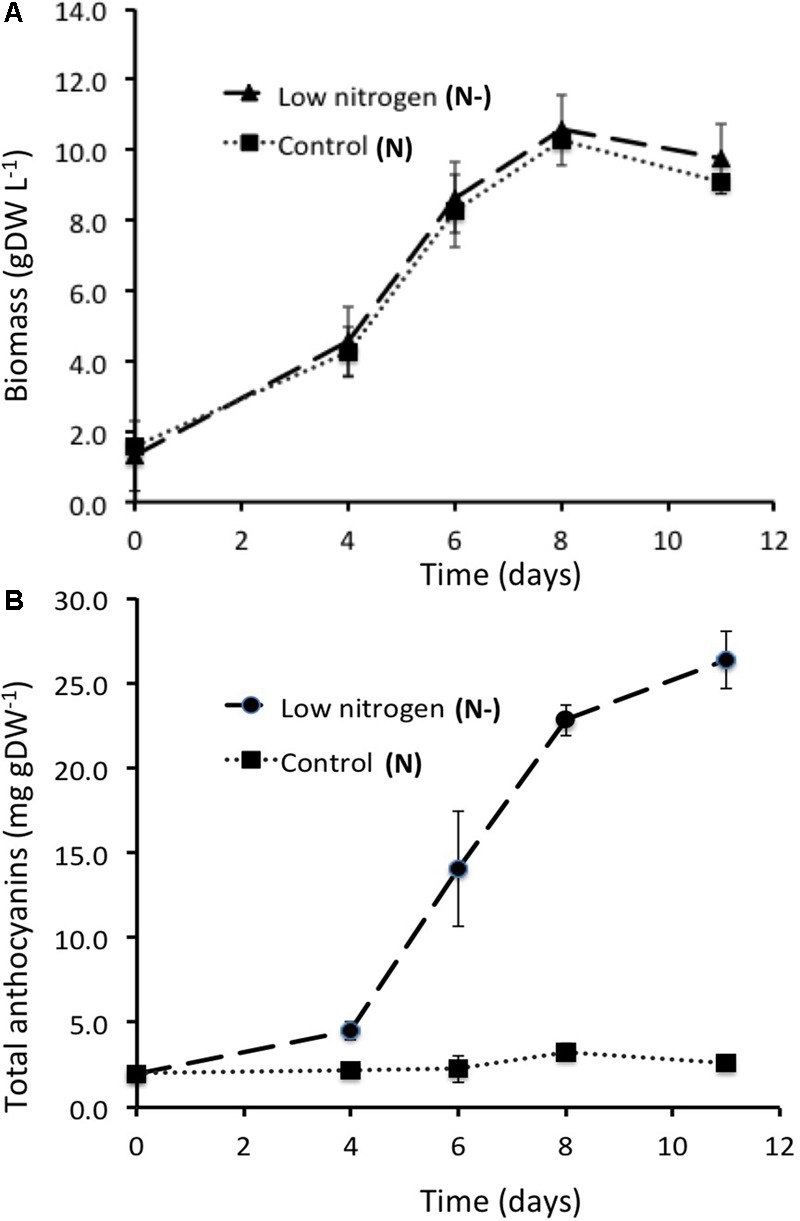
Cell growth of the GT3 **(A)** and the anthocyanin production **(B)** in control and low nitrogen conditions (N, control, 25 mM KNO_3_ and N–, nitrogen limitation, 5 mM KNO_3_, respectively). Data are the mean ± SE (*n* = 3) (exp. 1 and exp. 2).

Dry biomass accumulation kinetics were nearly identical for cells cultured in either control or low nitrogen conditions, increasing from about 1.6 g DW^-1^ of cells per L at culture initiation to about 10 g DW^-1^ at the 8th day of culture, and starting to decline afterwards (**Figure 2A**). Conversely, total anthocyanin accumulation patterns were strikingly different in control and low nitrogen condition cultures (**Figure 2B**). In control cells (N, 25 mM NO_3_^-^), total anthocyanin content was fairly stable during the culture (around 2–3 mg g DW^-1^), whereas in cells cultivated at low nitrogen (N-, 5 mM NO_3_^-^) total anthocyanin cell content strongly increased from the 4th day of culture to the 12th day, reaching a maximum of about 20 mg g DW^-1^ at the end of the culture.

This result has been reproduced with three cell cultures (named exp. 1, exp. 2, and exp. 3), that were conducted in the same conditions. Similar growth profiles were observed with about 10 g DW^-1^ of cells per L at the 11th day of culture (Supplementary Figure 1). Plotted in log-scale, the cell biomass concentration increased linearly up to the 6th day of culture. Maximal specific growth rate was reached at the 4th day of culture, in the exponential growth phase. Consequently, the steady state was assumed for modeling, i.e., the state when there was no accumulation of internal metabolites of the network, at day 4. Nevertheless, all data have also been collected and analyzed at the 6th day of culture to assess the evolution of the metabolic fluxes from day 4 to 6. For each experiment, the specific growth rate (μ in day^-1^) was determined as the growth rate (g DW day^-1^) related to the biomass (g DW) and the average was: 0.288 ± 0.013 day^-1^ and 0.160 ± 0.045 day^-1^ at day 4 and 6 of the culture, respectively.

### Nitrogen and Carbon Consumption in Culture Medium

Both total nitrogen and total carbon have been determined in the medium (for experiments 2 and 3, Supplementary Figure 2). Only in the case of low nitrogen condition (N-), nitrogen was limiting and even fully depleted since the 6th day of culture. The total carbon concentration in the medium was stable since the beginning up to the 8th day of culture, implying that there was no carbon limitation.

### Glucose and Fructose as Metabolized Sugars

Sucrose (20 g L^-1^) was the carbon source supplied in the culture medium, but it is generally cleaved to form hexoses by cell wall invertase activity (Atanassova et al., 2003; Chen et al., 2013). Measurements of sucrose, glucose, and fructose in the medium during 2 cultures (exp. 2, Supplementary Figure 3; exp. 3, Supplementary Figure 4) showed that hexose concentrations were higher than sucrose concentration at the 4th of culture. Thus glucose and fructose were assumed to be the main sugars metabolized by the cells.

### Metabolic Fluxes Modeling

#### Flux-Balance Model

The flux-balance model was constructed by integrating biochemical and physiological knowledge about the stoichiometry of reactions and the boundary conditions, i.e., the definition of external compounds. The model describes one cell and assumes that the suspension is homogeneous. The model combines the central metabolism previously described (Beurton-Aimar et al., 2011; Colombié et al., 2015) dedicated to breakdown and transformation of extracellular nutriments to produce energy and metabolic precursors (amino acids, proteins, cell wall, …) and the secondary metabolic pathway to produce the main polyphenols (anthocyanins, flavonols, tannins, and stilbenes). This network of reactions (schematized in Supplementary Figure 5, and the list of the stoichiometric reactions in Supplementary Table 1) includes the glycolysis, the tricarboxylic acid cycle (TCA), the pentose phosphate pathway, starch metabolism, and sucrose metabolism. The carbon source was described through glucose and fructose uptake (*Vglc-up, Vfru-up*). The inorganic nitrogen source was nitrate *(Vno3-up*) involving enzymes of the nitrogen assimilation pathway [nitrate reductase (*Vnr*), glutamine synthetase (*Vgs*), and glutamate synthase (*Vgogat*)]. Ammonium, with a low concentration (2 mM), was neglected as nitrogen source. For the phenolic pathway, three reversible reactions involving naringenin, dihydroquercetin, and leucocyanidin (*Vnar, Vdhq*, and *Vlcc*) and two irreversible reactions involving cinnamate and coumaroyl coenzyme A (*Vpal* and *Vcoum*) were connected to central metabolism via phenylalanine. The fluxes directed toward the main phenolic compounds, i.e., anthocyanins, flavonols, tannins, and stilbenes were described by four overall reactions (*Vanthoc, Vflav, Vtannins*, and *Vstilb*, respectively). The main biosynthetic processes were described with overall reactions: (1) cell wall polysaccharides from UDP-glucose (*Vcw*), (2) protein synthesis (*Vprotein*) according to the measured amino acid composition of proteins (Supplementary Table 2), (3) fatty acids synthesis (diacyl glycerol, *Vdag*) from pyruvate and trioses phosphate according to total fatty acid biomass measurement (Supplementary Table 3), and (4) nucleotides synthesis (DNA and RNA, *Vnucleotides*) from ribose-5-phosphate by using plant metabolic pathway databases^2^. All other accumulated compounds were described as a simple accumulation: (1) malate (*Vac-mal*), (2) soluble sugars, i.e., glucose (*Vac-glc*), fructose (*Vac-fru*), and sucrose (*Vac-suc*), and (3) four groups of free amino acids, glutamate (*Vac-Glu)*, aspartate (*Vac-Asp)*, alanine (*Vac-Ala)*, and phenylalanine (*Vac-Phe)*. It has been checked that no metabolites were excreted in the medium (data not shown).

Energy intermediates, both ATP and NAD(P)H, were explicitly taken into account. The cofactors NADP/NADPH were linked to biomass and the phenolic compounds production, and the cofactors NAD/NADH and FAD/FADH were linked to ATP synthesis via two essential reactions of oxidative phosphorylation (*Vnrj1* and *Vnrj2*), which are associated to the mitochondrial respiration. Recycling of AMP by adenylate kinase is described by *Vadk*. The portion of synthesized ATP that is not used for growth has been balanced by the model as an ATP hydrolyzing reaction (*Vnga-ATPm*) that physiologically represents cellular maintenance (Amthor, 2000). Finally, all the cofactors were defined as internal metabolites, which means that they were balanced, thus constraining the metabolic network not only through the carbon and nitrogen balance but also through the redox and energy status.

In summary, the model of the metabolic network describes the main growth components of the cell through a set of *n* reactions involving *m* metabolites whose *m*_int_ were internal metabolites. At steady-state, the mass balance equation is expressed by

(1) $$\frac{dX_{\text{int}}}{dt}=NV=0$$

With *X*_int_ the vector of *m*_int_ internal metabolites, $V={\left(v_{\text{i}}\right)}_{\text{i}=1\ldots n}^{t}$ the flux vector composed by the rates of *n* reactions of the network, and N = (n_ij_) _i=1…m_int_, j=1…n_ the stoichiometry matrix where *n*_ij_ is the stoichiometric coefficient of metabolite *x*_int,i_ in reaction *j*. To solve the system, a lower and an upper bound constrained each flux.

#### Constraints Limiting the Flux Space and Resolution

The first type of constraints applied to limit the flux space to flux directions was inferred from thermodynamic properties of reversibility or irreversibility. Thus, among the internal reactions of the metabolic network, 33 were irreversible as indicated by unidirectional arrows on Supplementary Figure 5, which meant that their lower bounds were set to zero.

The second type of constraints was the maximal enzyme capacities. Experimentally determined activities of enzymes of central metabolism and flavonoid pathway, considered as maximal enzyme capacities (converted in mmol g DW^-1^ day^-1^, Supplementary Table 4) were used to limit each corresponding flux in the metabolic network. The same values, but negative, were used as lower bounds of reversible enzymes. When the capacity of a given enzyme was not known, the bounds were set to infinity.

The third type of constraint concerned the respiration rate (see Section “Materials and Methods”). The sum of the two reactions of ATP synthesis by oxidative phosphorylation (*Vnrj1* and *Vnrj2*) was constrained by the respiration measurements: 3.54 ± 0.18 and 2.86 ± 0.24 mmol g DW^-1^ day^-1^ in control and low nitrogen conditions at day 4 of culture, respectively.

Finally, the essential constraints required to set up the system were the external fluxes, also called exchange fluxes. Assuming steady state, these 16 fluxes (rates) were calculated from experimental data (Supplementary Table 5) and used as both lower and upper bounds. Also, respiration rates (Supplementary Table 6) were used to constrained ATP synthesis fluxes.

**FIGURE 3:**
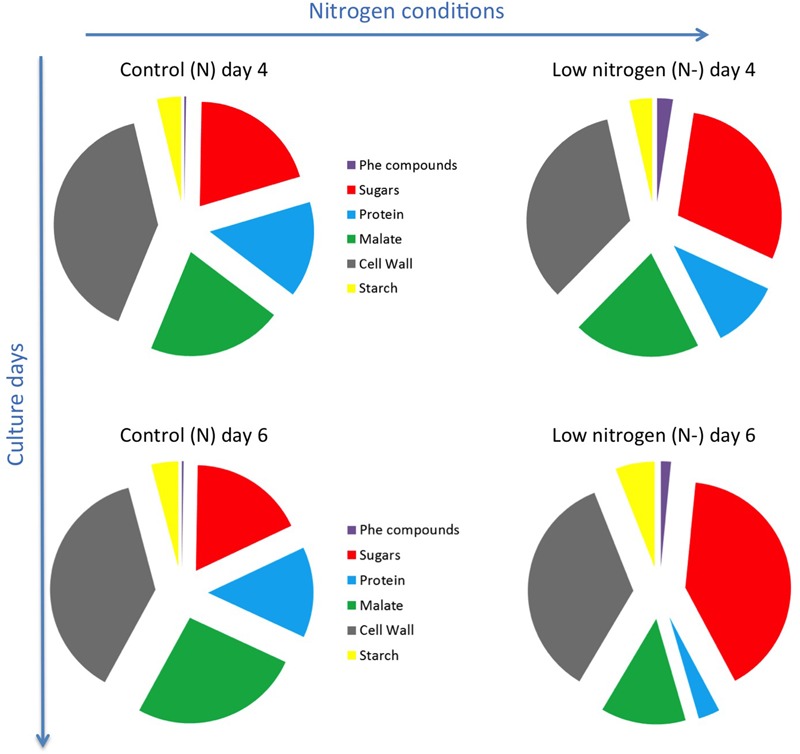
Schematic representation of biomass balance of GT3 cells at day 4 and 6 of culture. Phe: Phenolic; control conditions (N, 25 mM KNO_3_); low nitrogen (N–, 5 mM KNO_3_).

The mass balance of accumulated metabolites and biomass components covered an average of 81 and 91% of the dry biomass in control and nitrogen-limiting conditions, respectively (Supplementary Table 5). The accumulation of phenolic compounds in nitrogen-limiting condition was followed by an increase in sugar accumulation in cells at the expense of proteins synthesis and malate accumulation, especially at day 6 (**Figure 3**).

**FIGURE 4:**
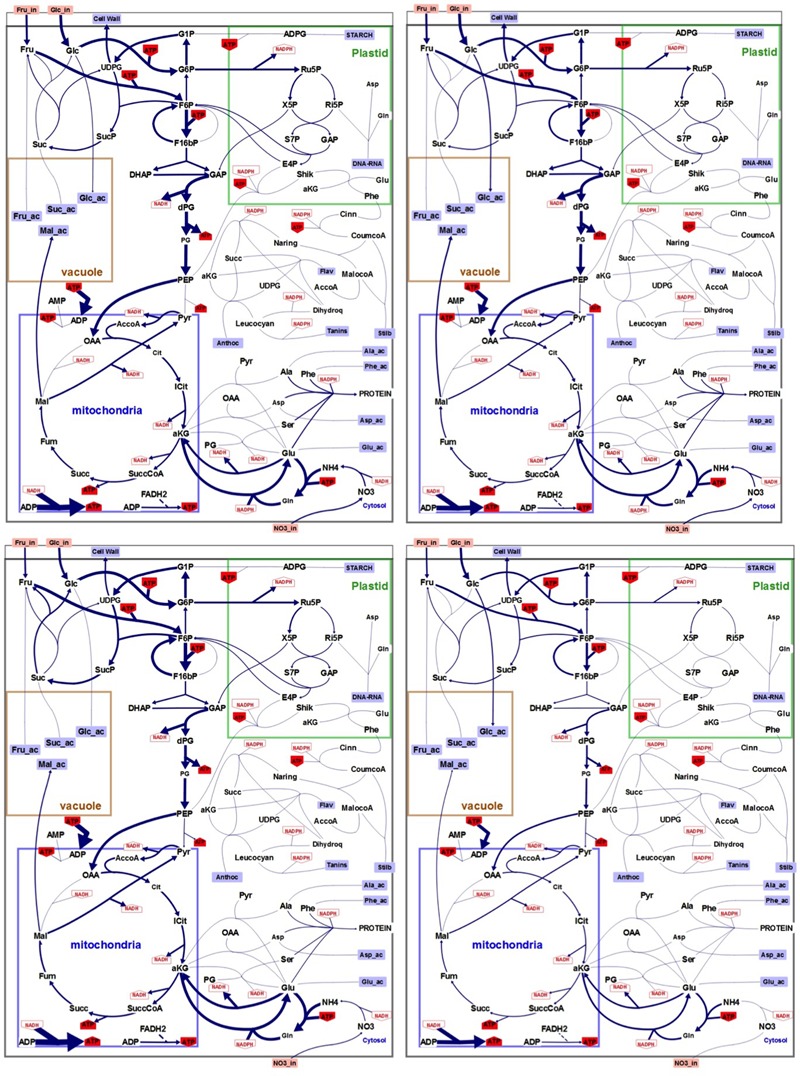
Flux maps generated through FBA modeling. Upper left panel: control conditions (N), day 4. Upper right panel: low nitrogen (N–), day 4. Lower left panel: control conditions (N), day 6. Lower right panel: (N–), day 6. Size of the arrows is proportional to flux absolute intensity. Imported metabolites are in orange boxes. External metabolites are in blue squares. NADH, NADPH, and ATP use by each reaction are in red. Irreversible reactions are indicated by unidirectional arrows. The compartmentation indicated on the figure is reminiscent of the physiological situation. Illustration was designed with the software Omix (Droste et al., 2011).

Flux minimization, which leads to a unique solution (Holzhutter, 2004), was used as the objective function to solve the system and generate flux maps in both N and N- conditions (**Figure 4**). Unsurprisingly, flux maps obtained with low nitrogen cultured cells compared to control condition showed higher fluxes in primary than in secondary metabolism both at 4 and 6 days of culture. Also a lower flux of ATP synthesis is pointed in low nitrogen condition.

**FIGURE 5:**
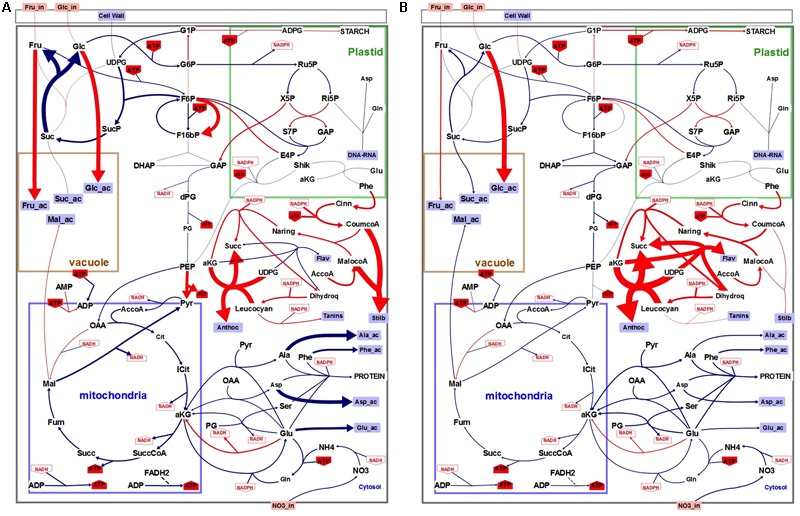
Schematic representation of the flux variations between control (N) and low (N–) nitrogen culture conditions at day 4 **(A)** or 6 **(B)**. Each color indicates relative flux variations between the two conditions: blue indicates decreased fluxes and red increased fluxes. Size of the arrows is proportional to flux variation intensity.

More than the absolute values of the calculated fluxes, we were interested in the relative changes in the fluxes to look for cell metabolism reprograming under low nitrogen condition (Supplementary Table 7 and **Figure 5**). Concerning external fluxes (4th day) the main changes in nitrogen-limiting condition were the increase in accumulation of phenolic compounds (except flavonols), hexoses and starch (**Figure 5A**). Conversely protein synthesis, sucrose accumulation, and respiration were decreased. Consequently, the calculated fluxes in the main pathways (glycolysis, TCA, PPP…) were decreased of about 20 to 30%, except the fluxes of the phenolic pathway which were increased: *Vmacl, Vshik, Vpal*, and *Vcoum* by 38% and, *Vnar, Vdhq*, and *Vlcc* by 26%. More surprisingly, two internal fluxes, the PPi-dependent phosphofructokinase (*Vpfp*) and the pyruvate kinase (*Vpk*) were strongly increased (80 and 63%), and also glucose and fructose uptakes, were also slightly increased (7 and 6%) at day 4. Finally, fluxes corresponding to enzymatic steps of the flavonoid biosynthesis that use α-ketoglutarate (α-KG) as a reducing agent and convert it to succinate, namely *Vdhq* and *Vanthoc* were increased by 25 and 104%, also at day 4. Conversely, α-KG conversion to succinate the TCA cycle (*Vkgdh*) was decreased by 25% in low nitrogen culture conditions.

The global behavior of external fluxes was exacerbated at day 6 and resulted in similar observations than at day 4, i.e., a global diminution of all fluxes (40–50%) but here without significant change in *Vpfp* and *Vpk* and sugar uptake (**Figure 5B** and Supplementary Table 7). Changes in fluxes for *Vdhq* and *Vanthoc* were further enhanced by low nitrogen culture conditions, compared to control, with an increase of 129 and 518%, respectively. A third α-KG-dependent flavonoid biosynthetic flux was also strongly enhanced by 320%. TCA-linked metabolic flux that converts α-KG into succinate (*Vkgdh*) was reduced by 50%.

**FIGURE 6:**
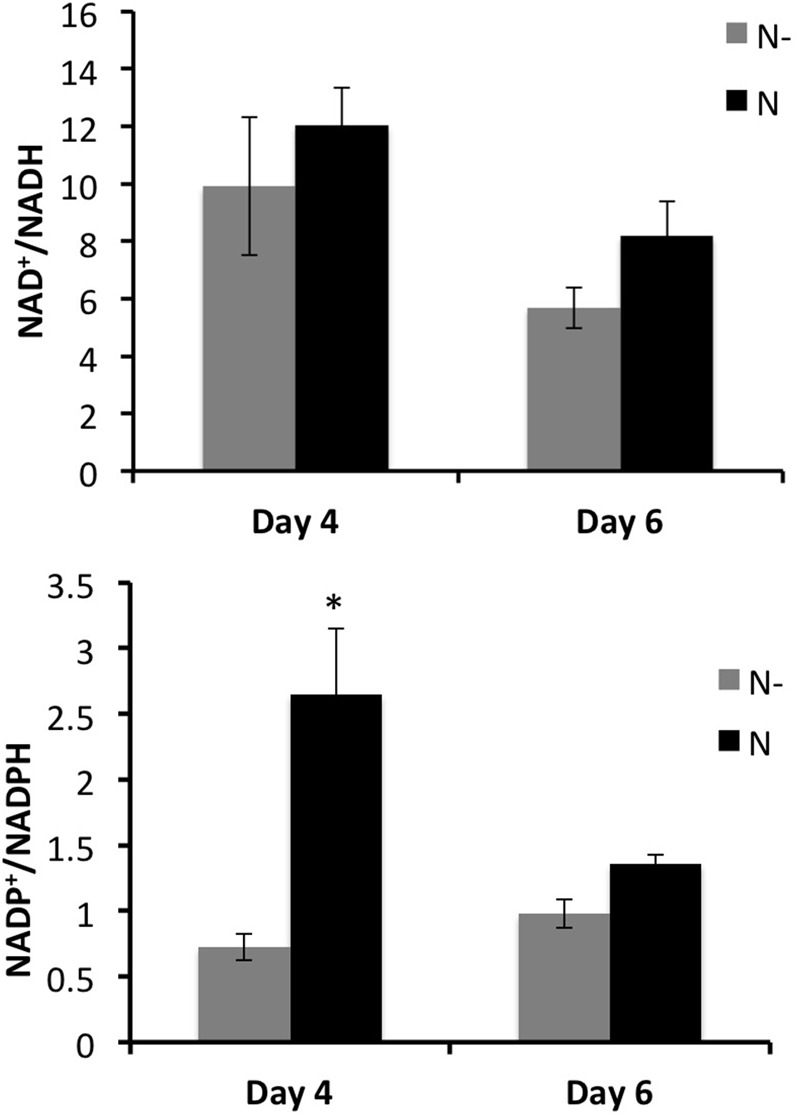
Measured NAD^+^/NADH and NADP^+^/NADPH ratios in GT3 cell suspension, for two nitrogen conditions (N–, gray bars and N, black bars) at 4 and 6 days of culture. Values are means ± SE of 6 replicates (exp. 1 and exp. 2) and ^∗^indicates significant difference according to Student’s *t*-test with *p* < 0.05.

The internal metabolite concentrations were not accessible with the flux-balance model. Then complementary analyses have been done to determine the total contents of redox metabolism coenzymes (NAD^+^, NADH, NADP^+^, and NADPH). While NADH was slightly affected, NADPH significantly increased in low nitrogen condition compared to the control (Supplementary Table 8). Thus, the NADP^+^/NADPH ratio was significantly lower in low nitrogen condition at day 4 (**Figure 6**). The same trend was observed at day 6, but was not deemed statistically significant according to Student’s *t*-test (**Figure 6**). These results clearly showed an excess of NADPH, concomitant with the accumulation of anthocyanin (at day 4 and 6) and stilbene compounds (at day 4).

## Discussion

### Low Nitrogen Stimulates Anthocyanin Biosynthesis in GT3 Grapevine Cells

Under nitrogen limitation, an increase of the total anthocyanin content, especially peonidin and petunidin derivatives was noted. Moreover, the low phenylalanine concentration in cells cultivated in limiting nitrogen condition is in agreement with an increase in the phenylpropanoid catabolic flux, supported by the increase in PAL activity. Stimulation of anthocyanin biosynthesis in vineyard-grown grape berries by low nitrogen availability has been well documented in the literature, with an increase of around 30% in berry anthocyanin content (see for example Keller and Hrazdina, 1998; Hilbert et al., 2003; Soubeyrand et al., 2014). Similar studies using grapevine cell suspensions are much more scarce, however. In our experiments, GT3 grapevine cell suspension responded to low nitrogen in the culture media by a c.a. 700%, in average, increase total anthocyanin accumulation. This result is in the same order of magnitude as the results previously obtained on strawberry (Mori and Sakurai, 1994), or Gamay Fréaux grapevine (Do and Cormier, 1991), thus validating the GT3 cell suspension culture used in this work for acquiring the dataset that allowed us to perform FBA modeling.

### Building a FBA Model That Links Central Primary Metabolism and the Polyphenol Secondary Metabolism

The metabolic model utilized in this work was sufficiently detailed to describe the global functioning of the cell. The originality of this work was to couple both primary and secondary metabolism, including the flavonoid biosynthetic pathway. As far as we know, in plant science only few models take into account secondary metabolic pathways. A genome-scale metabolic model of maize has already been reconstructed (Saha et al., 2011). Bekaert et al. (2012) described their updated mathematical model of *Arabidopsis thaliana* Columbia metabolism, which adds the glucosinolates, an important group of secondary metabolites, to the reactions of primary metabolism. In a recent review, Collakova et al. (2012) showed that metabolism can be modeled mathematically by using models and genome-scale models (GEMs) predicting the combination of flux values of a defined metabolic network given the influence of internal and external signals. Nevertheless plant GEMs tend to be accurate in predicting only qualitative changes in selected aspects of central carbon metabolism, while secondary metabolism is largely neglected mainly due to the missing (unknown) genes and metabolites. As such, these models are suitable for exploring metabolism in simplified models such cell cultures in plants grown in favorable (controlled) conditions, but not in field-grown plants that have to cope with environmental changes in complex ecosystems (Collakova et al., 2012).

### Cell Energy and Reducing Power as a Driver for Anthocyanin Biosynthesis in Grape Cells

The question of the existence of a metabolic driver that would fuel the increase in anthocyanin (and more generally in flavonoid) biosynthesis in such situation remains open. One emerging property of the FBA-generated flux maps is the fact that in low nitrogen conditions (N-), several enzymatic steps that consume ATP and reducing power (NADPH or NADH) have their metabolic flux either maintained or increased (i.e., starch synthase, phosphofructokinase, enzymes of the pentose-phosphate pathway, all the enzymes of the phenylpropanoid and flavonoid biosynthetic pathway, as well as the stilbene biosynthetic pathway). Conversely, most of the metabolic fluxes that lead to ATP, NADH, or NADPH formation were decreased by low nitrogen conditions (i.e., phosphoglycerate kinase and pyruvate kinase in the lower part of the glycolytic pathway, the malic enzyme, most of the TCA cycle enzymes with noticeable exception of the malate dehydrogenase which has its metabolic flux slightly increased). This strongly advocates for a link between cell energy status (i.e., excess of ATP and reducing power) and secondary metabolism, confirming an hypothesis made by Hernández and Van Breusegem (2010). Recently, redox-dependent modulation of the anthocyanin pathway has been reported in Arabidopsis leaves during exposure to high light intensity (Page et al., 2011; Viola et al., 2016), or in Citrus callus (Cao et al., 2015). FBA modeling results strongly support that hypothesis, as well as actual NADP^+^/NADPH ratio measurements, pointing toward ATP and NADPH excess as a metabolic driver for flavonoid (and particularly anthocyanin) biosynthesis in grapevine GT3 cells. In the same review from Hernández and Van Breusegem (2010) also hypothesized that flavonoid biosynthesis could also constitute a carbon sink in situations of high carbon-to-nutrient ratio. Indeed in leaves from plants such as Rosemary or Tea trees, flux analysis suggests that up 20% of the fixed carbon would flow through the phenylpropanoid pathway, leading to a phenolic content accounting for up to 30% of dry matter, making it the main non-structural carbon sink of the plant, and thus an efficient mechanism to deal with carbon excess, without mobilizing any nitrogen (Hernández et al., 2004; Rippert et al., 2004; Yao et al., 2005). In the case of GT3 grape cells, however, model flux calculations and biomass composition analysis demonstrated that anthocyanins, and more broadly flavonoids, represent only a marginal storage sink for non-structural carbon (0.49 and 1.5% of total dry matter, at day 4 and 6, respectively, in N- condition), ruling out a role of anthocyanin (and more broadly flavonoids) biosynthesis as an effective mechanism for coping with carbon excess in high carbon to nitrogen ratio situations. Instead, FBA model output and biomass analysis indicate that carbon excess is dealt with by diverting embolic flux to vacuolar storage of soluble sugars (hexose and to a lesser extent sucrose) and malic acid. This discrepancy could be linked to fact that cell suspensions and whole organs such as leaves obviously differ in their behavior in term of carbon management. Leaves can act both as source and sinks for carbon, whether cultured cells only acts as carbon sink. The comparison is thus limited, but nevertheless points out two potentially different strategies for leaves and grape cells to cope with carbon excess.

### α-Ketoglutarate Levels as a Potential Regulator of Anthocyanin Biosynthesis in Grape Cells

Besides the fact that low nitrogen culture conditions might lead to an altered cell energy status (i.e., an excess of ATP and NADPH), another output of the FBA-generated flux maps is that three fluxes of the flavonoid pathway that use α-KG were strongly up-regulated in low nitrogen cultured cells. α-KG has emerged in the past decade as a signal molecule in plants, linking TCA cycle to secondary metabolism, including the flavonoid pathway (Araújo et al., 2014). Indeed, three enzymatic steps of the flavonoid pathway use α-KG as reducing agent in their catalytic cycle: the flavanone 3β-hydroxylase, the flavonol synthase and the anthocyanidin synthase (Turnbull et al., 2004). Under low nitrogen culture conditions, consumption of α-KG by GOGAT for glutamate synthesis is bound to decreased. This is advocated by model output that predicts a diminution of 21 and 50% at day 4 and 6, respectively, potentially leading to an increase of cell α-KG level, which would be used to fuel anthocyanin and more generally flavonoid biosynthesis. Thus, α-KG availability would be part of the metabolic driver that lead to enhanced flavonoid biosynthesis high carbon-to-nitrogen ratio conditions. Actual α-KG level measurements would be required to further advocate this hypothesis.

## Conclusion

Flux balance analysis modeling was used to investigate metabolic flux reprogramming in grapevine cells in response to low nitrogen culture conditions and revise the well-known up-regulation of anthocyanin biosynthesis in response to low nitrogen availability. Model outputs unambiguously point toward cell energy excess and increased α-KG availability as the metabolic drivers of anthocyanin synthesis (and more broadly flavonoid synthesis) under high carbon-to-nitrogen ratio conditions. This work was conducted in a cell suspension culture, and the next obvious question is whether such a metabolic driver effect is also occurring in ripening berries of red grape varieties, which accumulate anthocyanins to high levels in their exocarp cells, a key feature for high quality red wine making. Further modeling and biochemical work is needed to address that question.

## Author Contributions

ES, GH, CR, StC, LM-P, and BB performed the experiments and the analytical work. SoC performed model construction and calculations, participated to data analysis and manuscript writing. MD-N generated the flux maps. YG, J-MM, ZD, and SD discussed the results and performed manuscript critical reading. EG led the project and designed the experimental flowchart, discussed the results and coordinated the manuscript writing and critical reading.

## Conflict of Interest Statement

The authors declare that the research was conducted in the absence of any commercial or financial relationships that could be construed as a potential conflict of interest.

## Footnotes

1 http://www.bibs.inra.fr

2 http://www.plantcyc.org
