# Supplementary material for: Constraint-Based Modeling Highlights Cell Energy, Redox Status and α-Ketoglutarate Availability as Metabolic Drivers for Anthocyanin Accumulation in Grape Cells Under Nitrogen Limitation
Source: Front Plant Sci. 2018 May 17;9:421. doi: 10.3389/fpls.2018.00421 (PMC5966944; doi:10.3389/fpls.2018.00421)
Supplement: Supplementary file 2 [file Presentation_1.PDF]

SUPPLEMENTAL FIGURES

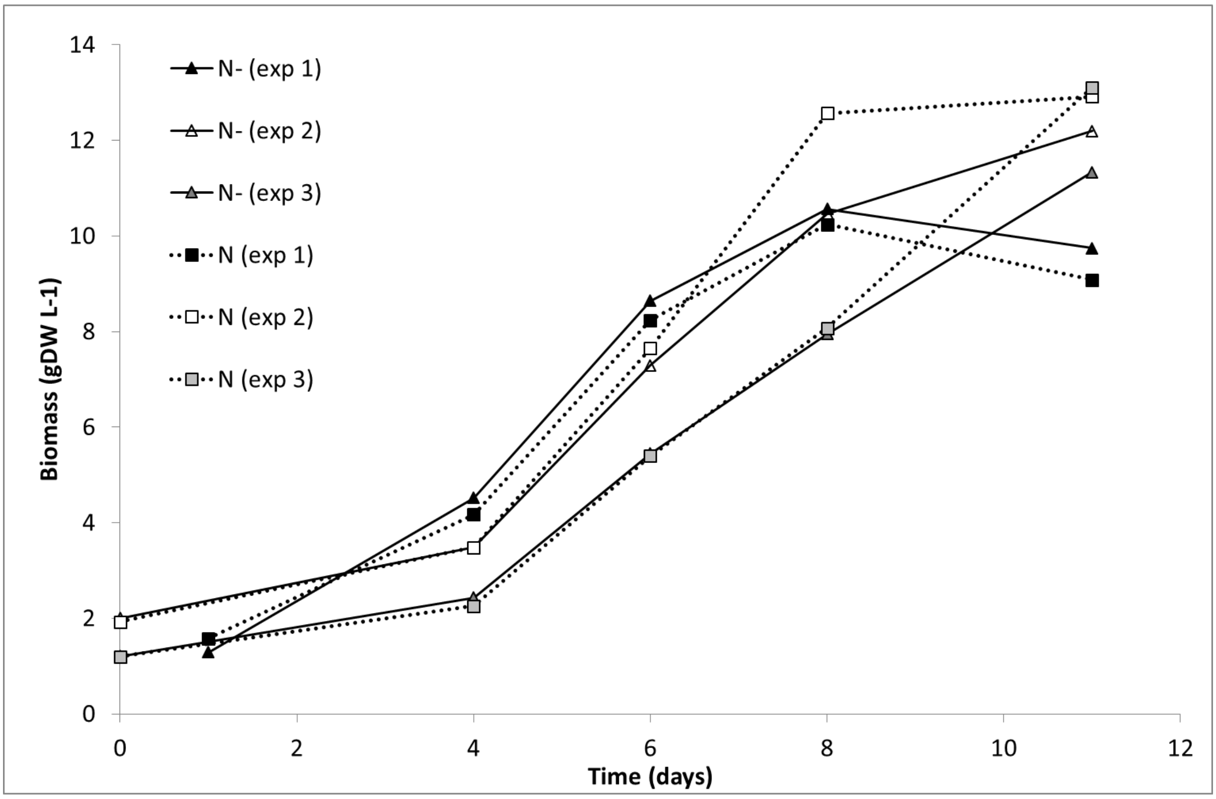

Supplemental Figure 1. Growth curves of 3 GT3 cells (exp1, exp2 and exp3) in control (N, 25 mM KNO_3) and low nitrogen (N-, 5 mM KNO_3) conditions, versus culture time (days of culture).

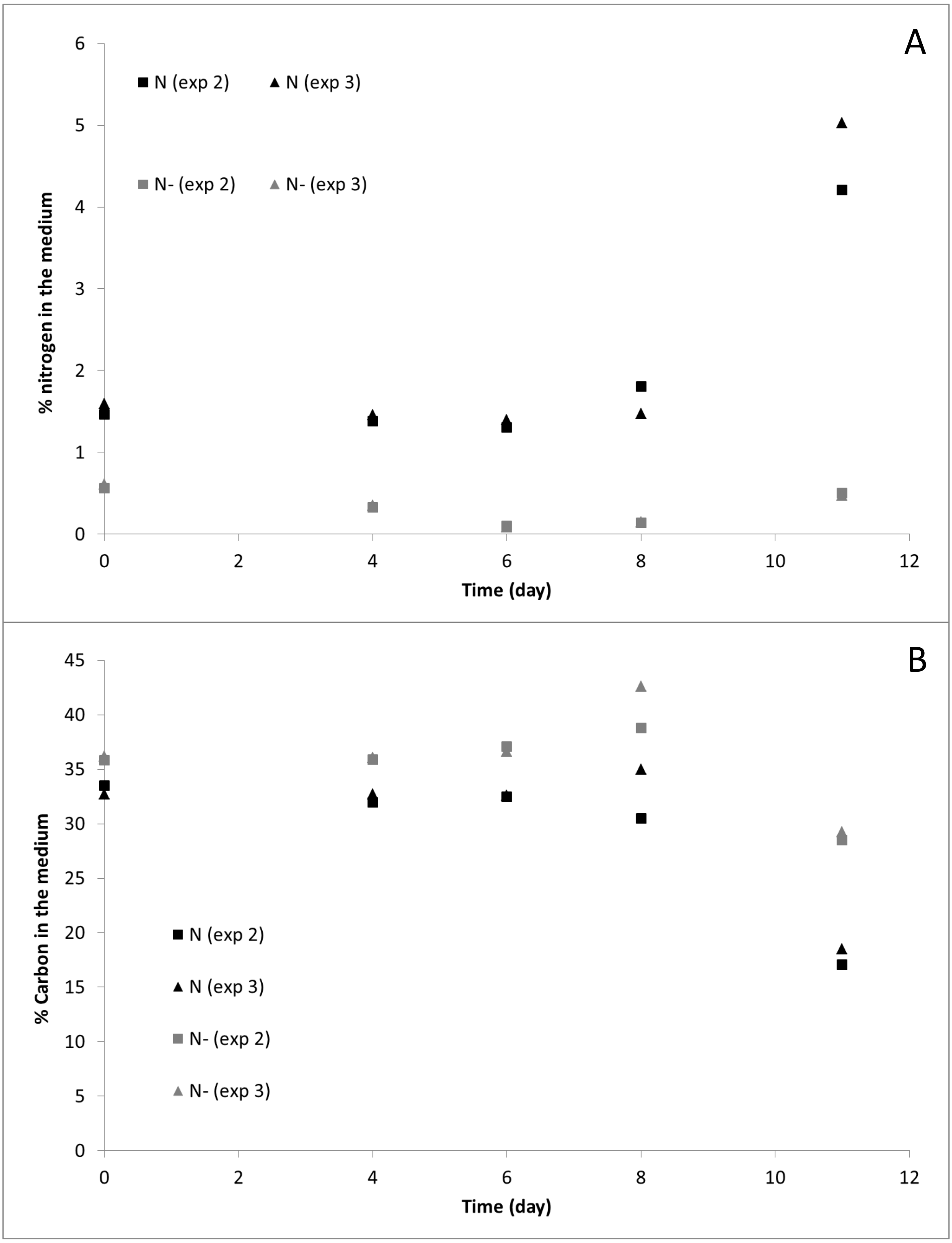

Supplemental Figure 2: Time course of the total nitrogen (A) and carbon (B) content in the medium, determined for two GT3 cell cultures (exp2 and exp3) in control (N, 25 mM KNO_3) and low nitrogen conditions (N-, 5 mM KNO_3).

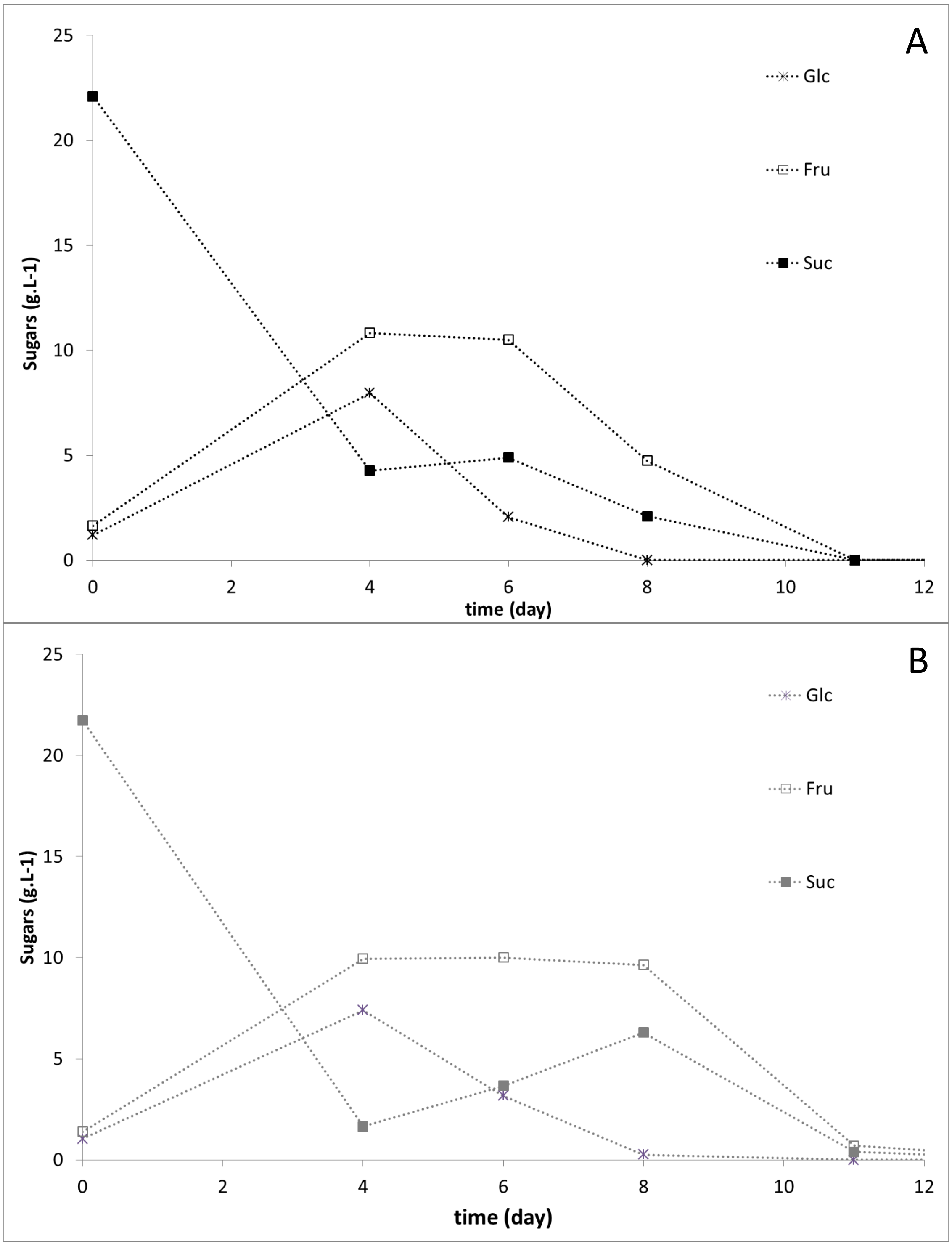

Supplemental Figure 3: Time course of glucose, fructose and sucrose content evolution in GT3 cell culture for exp2 in control (A) and low nitrogen (B) conditions (25 mM and 5 mM KNO3 respectively). Glc: glucose; Fru: fructose; Suc: sucrose.

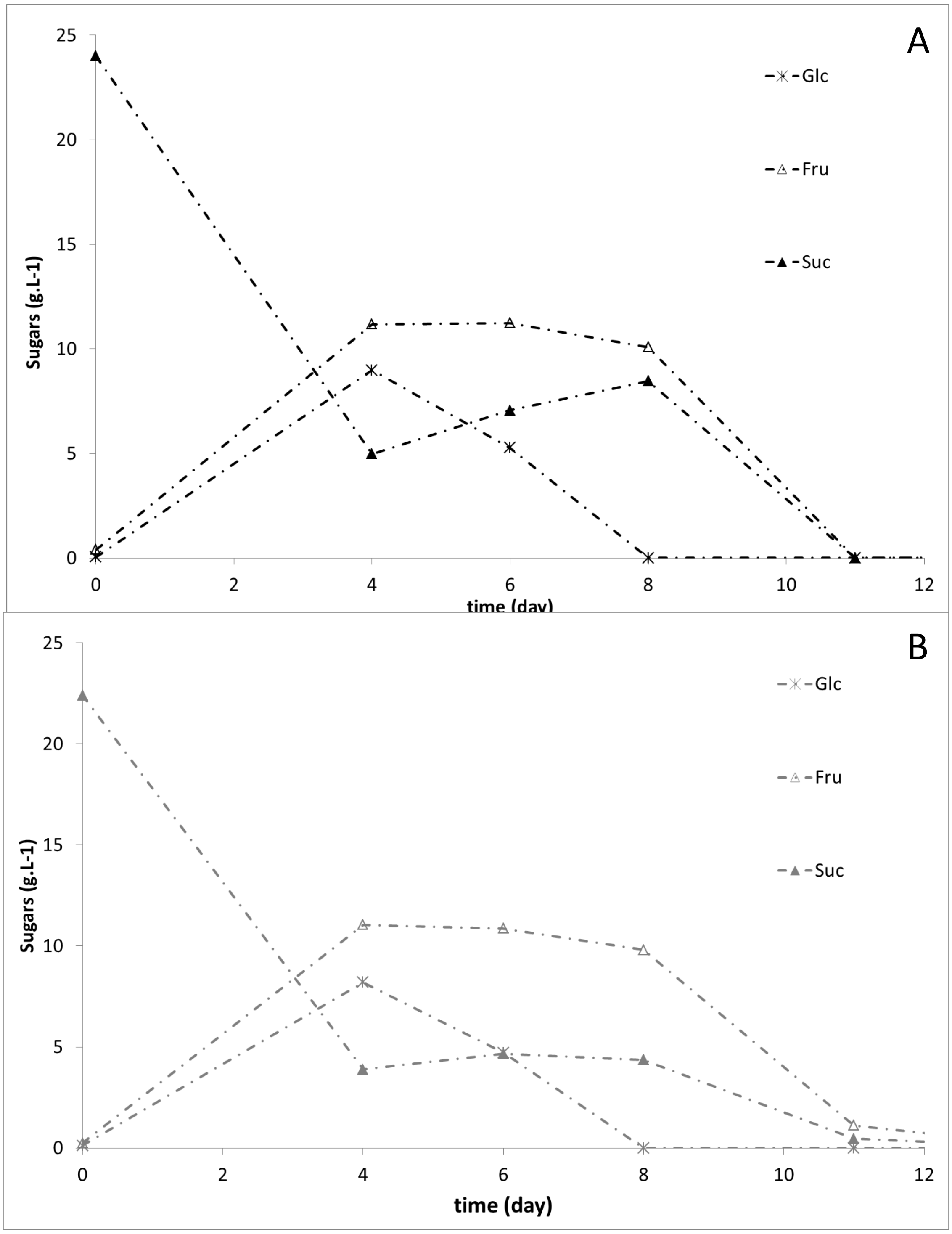

Supplemental Figure 4: Time course of glucose, fructose and sucrose content evolution in GT3 cell culture for exp3 in control (A) and low nitrogen (B) conditions (25 mM and 5 mM KNO3 respectively). Glc: glucose; Fru: fructose; Suc: sucrose.

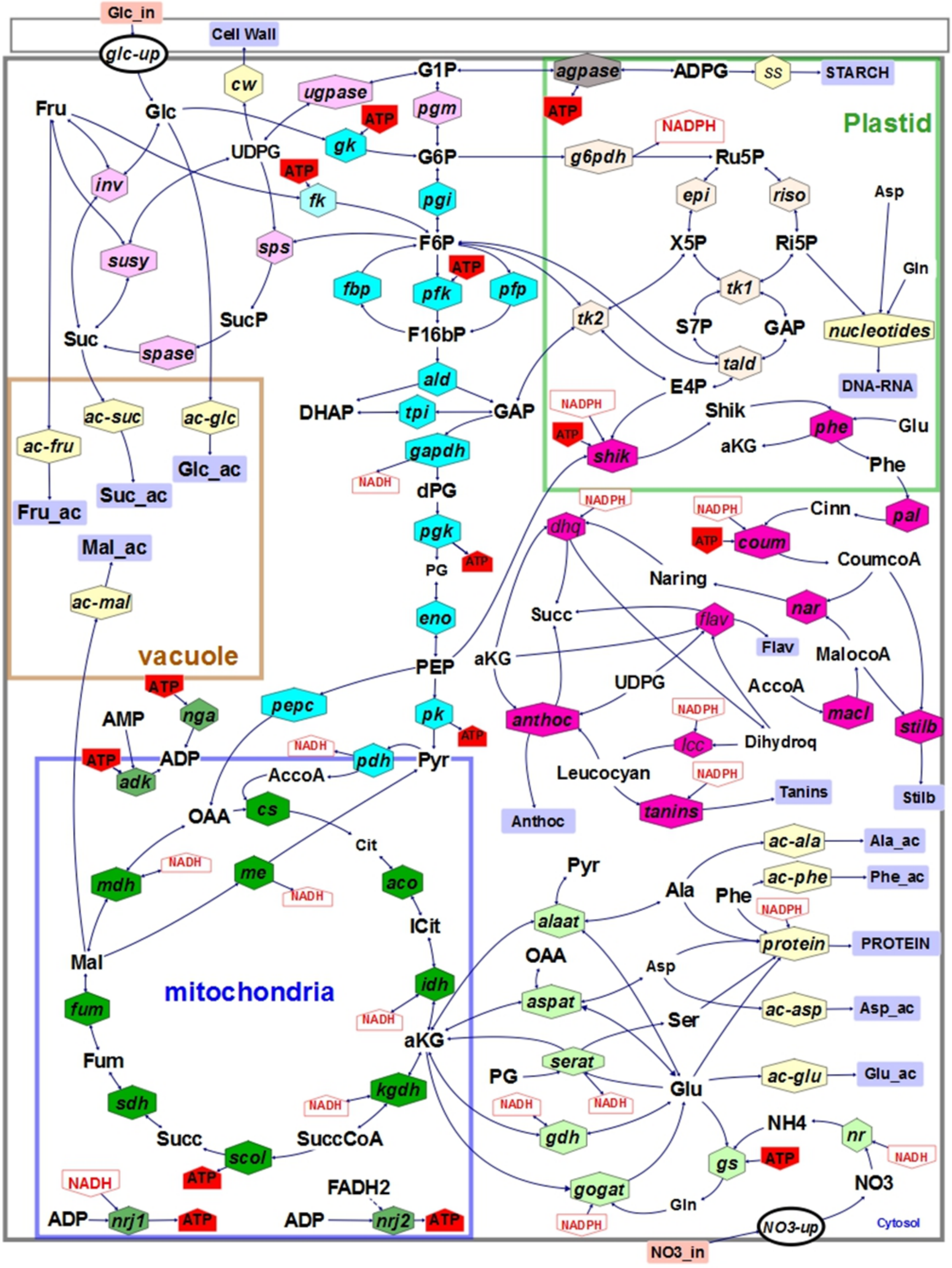

Supplemental Figure 5: Structure of the metabolic network. Each color indicates a pathway: pink for the sucrose synthesis, blue for the glycolysis, orange for the pentose phosphate pathway, dark green for the TCA cycle, light green for the nitrogen assimilation and purple

for the phenylalanine synthesis and phenylpropanoid pathway. Imported metabolites are in orange boxes. External metabolites are in blue square. NADH, NADPH and ATP use by each reaction are in red. Irreversible reactions are indicated by unidirectional arrows. The compartmentation indicated on the figure is reminiscent of the physiological situation. Illustration designed with the software Omix (Droste et al., 2011).
