# Supplementary material for: Constraint-Based Modeling Highlights Cell Energy, Redox Status and α-Ketoglutarate Availability as Metabolic Drivers for Anthocyanin Accumulation in Grape Cells Under Nitrogen Limitation
Source: Front Plant Sci. 2018 May 17;9:421. doi: 10.3389/fpls.2018.00421 (PMC5966944; doi:10.3389/fpls.2018.00421)
Supplement: Supplementary file 3 [file Presentation_2.pdf]

Metabolic model of the grape cells in *smbl*


```
<sbml xmlns="http://www.sbml.org/sbml/level2" level="2" version="1">
   <model id="WineCells_pub" name="WineCells_pub">
      <listOfCompartments>
         <compartment id="External_Species"/>
         <compartment id="Internal_Species"/>
      </listOfCompartments>
      <listOfSpecies>
         <species id="ADPG" name="ADPG" boundaryCondition="false"
compartment="Internal_Species"/>
         <species id="aKG" name="aKG" boundaryCondition="false"
compartment="Internal_Species"/>
         <species id="Ala" name="Ala" boundaryCondition="false"
compartment="Internal_Species"/>
         <species id="Asp" name="Asp" boundaryCondition="false"
compartment="Internal_Species"/>
         <species id="AcCoA" name="AcCoA" boundaryCondition="false"
compartment="Internal_Species"/>
         <species id="Cit" name="Cit" boundaryCondition="false"
compartment="Internal_Species"/>
         <species id="DHAP" name="DHAP" boundaryCondition="false"
compartment="Internal_Species"/>
         <species id="dPG" name="dPG" boundaryCondition="false"
compartment="Internal_Species"/>
         <species id="E4P" name="E4P" boundaryCondition="false"
compartment="Internal_Species"/>
         <species id="F16bP" name="F16bP" boundaryCondition="false"
compartment="Internal_Species"/>
         <species id="F6P" name="F6P" boundaryCondition="false"
compartment="Internal_Species"/>
         <species id="Fru" name="Fru" boundaryCondition="false"
compartment="Internal_Species"/>
         <species id="Fum" name="Fum" boundaryCondition="false"
compartment="Internal_Species"/>
         <species id="G1P" name="G1P" boundaryCondition="false"
compartment="Internal_Species"/>
         <species id="G6P" name="G6P" boundaryCondition="false"
compartment="Internal_Species"/>
         <species id="GAP" name="GAP" boundaryCondition="false"
compartment="Internal_Species"/>
         <species id="Glc" name="Glc" boundaryCondition="false"
compartment="Internal_Species"/>
         <species id="Gln" name="Gln" boundaryCondition="false"
compartment="Internal_Species"/>
         <species id="Glu" name="Glu" boundaryCondition="false"
compartment="Internal_Species"/>
         <species id="ICit" name="ICit" boundaryCondition="false"
compartment="Internal_Species"/>
         <species id="Mal" name="Mal" boundaryCondition="false"
compartment="Internal_Species"/>
```

```
        <species id="OAA" name="OAA" boundaryCondition="false"
compartment="Internal_Species"/>
        <species id="PEP" name="PEP" boundaryCondition="false"
compartment="Internal_Species"/>
        <species id="Pyr" name="Pyr" boundaryCondition="false"
compartment="Internal_Species"/>
        <species id="PG" name="PG" boundaryCondition="false"
compartment="Internal_Species"/>
        <species id="Ri5P" name="Ri5P" boundaryCondition="false"
compartment="Internal_Species"/>
        <species id="Ru5P" name="Ru5P" boundaryCondition="false"
compartment="Internal_Species"/>
        <species id="S7P" name="S7P" boundaryCondition="false"
compartment="Internal_Species"/>
        <species id="Ser" name="Ser" boundaryCondition="false"
compartment="Internal_Species"/>
        <species id="Suc" name="Suc" boundaryCondition="false"
compartment="Internal_Species"/>
        <species id="Succ" name="Succ" boundaryCondition="false"
compartment="Internal_Species"/>
        <species id="SuccCoA" name="SuccCoA" boundaryCondition="false"
compartment="Internal_Species"/>
        <species id="SucP" name="SucP" boundaryCondition="false"
compartment="Internal_Species"/>
        <species id="UDPG" name="UDPG" boundaryCondition="false"
compartment="Internal_Species"/>
        <species id="X5P" name="X5P" boundaryCondition="false"
compartment="Internal_Species"/>
        <species id="NAD" name="NAD" boundaryCondition="false"
compartment="Internal_Species"/>
        <species id="NADH" name="NADH" boundaryCondition="false"
compartment="Internal_Species"/>
        <species id="NADP" name="NADP" boundaryCondition="false"
compartment="Internal_Species"/>
        <species id="NADPH" name="NADPH" boundaryCondition="false"
compartment="Internal_Species"/>
        <species id="AMP" name="AMP" boundaryCondition="false"
compartment="Internal_Species"/>
        <species id="ATP" name="ATP" boundaryCondition="false"
compartment="Internal_Species"/>
        <species id="ADP" name="ADP" boundaryCondition="false"
compartment="Internal_Species"/>
        <species id="UDP" name="UDP" boundaryCondition="false"
compartment="Internal_Species"/>
        <species id="UTP" name="UTP" boundaryCondition="false"
compartment="Internal_Species"/>
        <species id="FAD" name="FAD" boundaryCondition="false"
compartment="Internal_Species"/>
        <species id="FADH2" name="FADH2" boundaryCondition="false"
compartment="Internal_Species"/>
        <species id="PPi" name="PPi" boundaryCondition="false"
compartment="Internal_Species"/>
```

```
        <species id="NH4" name="NH4" boundaryCondition="false"
compartment="Internal_Species"/>
        <species id="MaloCoA" name="MaloCoA" boundaryCondition="false"
compartment="Internal_Species"/>
        <species id="Shik" name="Shik" boundaryCondition="false"
compartment="Internal_Species"/>
        <species id="Phe" name="Phe" boundaryCondition="false"
compartment="Internal_Species"/>
        <species id="Cinn" name="Cinn" boundaryCondition="false"
compartment="Internal_Species"/>
        <species id="CoumCoA" name="CoumCoA" boundaryCondition="false"
compartment="Internal_Species"/>
        <species id="Naring" name="Naring" boundaryCondition="false"
compartment="Internal_Species"/>
        <species id="Dihydroq" name="Dihydroq"
boundaryCondition="false" compartment="Internal_Species"/>
        <species id="Leucocyan" name="Leucocyan"
boundaryCondition="false" compartment="Internal_Species"/>
        <species id="NO3" name="NO3" boundaryCondition="false"
compartment="Internal_Species"/>
        <species id="Glc_in" name="Glc_in" boundaryCondition="true"
compartment="External_Species"/>
        <species id="Fru_in" name="Fru_in" boundaryCondition="true"
compartment="External_Species"/>
        <species id="NO3_in" name="NO3_in" boundaryCondition="true"
compartment="External_Species"/>
        <species id="Anthoc" name="Anthoc" boundaryCondition="true"
compartment="External_Species"/>
        <species id="Flav" name="Flav" boundaryCondition="true"
compartment="External_Species"/>
        <species id="Stilb" name="Stilb" boundaryCondition="true"
compartment="External_Species"/>
        <species id="Tanins" name="Tanins" boundaryCondition="true"
compartment="External_Species"/>
        <species id="CW" name="CW" boundaryCondition="true"
compartment="External_Species"/>
        <species id="Glu_ac" name="Glu_ac" boundaryCondition="true"
compartment="External_Species"/>
        <species id="Asp_ac" name="Asp_ac" boundaryCondition="true"
compartment="External_Species"/>
        <species id="Ala_ac" name="Ala_ac" boundaryCondition="true"
compartment="External_Species"/>
        <species id="Phe_ac" name="Phe_ac" boundaryCondition="true"
compartment="External_Species"/>
        <species id="PROTEIN" name="PROTEIN" boundaryCondition="true"
compartment="External_Species"/>
        <species id="Mal_ac" name="Mal_ac" boundaryCondition="true"
compartment="External_Species"/>
        <species id="Glc_ac" name="Glc_ac" boundaryCondition="true"
compartment="External_Species"/>
        <species id="Fru_ac" name="Fru_ac" boundaryCondition="true"
compartment="External_Species"/>
```

```
          <species id="Suc_ac" name="Suc_ac" boundaryCondition="true"
compartment="External_Species"/>
          <species id="STARCH" name="STARCH" boundaryCondition="true"
compartment="External_Species"/>
          <species id="ADNARN" name="ADNARN" boundaryCondition="true"
compartment="External_Species"/>
          <species id="CO2" name="CO2" boundaryCondition="true"
compartment="External_Species"/>
       </listOfSpecies>
       <listOfReactions>
          <reaction id="Vpgi" reversible="true">
             <listOfReactants>
                <speciesReference species="G6P" stoichiometry="1"/>
             </listOfReactants>
             <listOfProducts>
                <speciesReference species="F6P" stoichiometry="1"/>
             </listOfProducts>
          </reaction>
          <reaction id="Vald" reversible="true">
             <listOfReactants>
                <speciesReference species="F16bP" stoichiometry="1"/>
             </listOfReactants>
             <listOfProducts>
                <speciesReference species="DHAP" stoichiometry="1"/>
                <speciesReference species="GAP" stoichiometry="1"/>
             </listOfProducts>
          </reaction>
          <reaction id="Vtpi" reversible="true">
             <listOfReactants>
                <speciesReference species="DHAP" stoichiometry="1"/>
             </listOfReactants>
             <listOfProducts>
                <speciesReference species="GAP" stoichiometry="1"/>
             </listOfProducts>
          </reaction>
          <reaction id="Vgapdh" reversible="true">
             <listOfReactants>
                <speciesReference species="GAP" stoichiometry="1"/>
                <speciesReference species="NAD" stoichiometry="1"/>
             </listOfReactants>
             <listOfProducts>
                <speciesReference species="dPG" stoichiometry="1"/>
                <speciesReference species="NADH" stoichiometry="1"/>
             </listOfProducts>
          </reaction>
          <reaction id="Vpgk" reversible="true">
             <listOfReactants>
                <speciesReference species="dPG" stoichiometry="1"/>
                <speciesReference species="ADP" stoichiometry="1"/>
             </listOfReactants>
             <listOfProducts>
                <speciesReference species="PG" stoichiometry="1"/>
```

```
      <speciesReference species="ATP" stoichiometry="1"/>
    </listOfProducts>
  </reaction>
  <reaction id="Veno" reversible="true">
    <listOfReactants>
      <speciesReference species="PG" stoichiometry="1"/>
    </listOfReactants>
    <listOfProducts>
      <speciesReference species="PEP" stoichiometry="1"/>
    </listOfProducts>
  </reaction>
  <reaction id="Vaco" reversible="true">
    <listOfReactants>
      <speciesReference species="Cit" stoichiometry="1"/>
    </listOfReactants>
    <listOfProducts>
      <speciesReference species="ICit" stoichiometry="1"/>
    </listOfProducts>
  </reaction>
  <reaction id="Vidh" reversible="true">
    <listOfReactants>
      <speciesReference species="ICit" stoichiometry="1"/>
      <speciesReference species="NAD" stoichiometry="1"/>
    </listOfReactants>
    <listOfProducts>
      <speciesReference species="aKG" stoichiometry="1"/>
      <speciesReference species="NADH" stoichiometry="1"/>
      <speciesReference species="CO2" stoichiometry="1"/>
    </listOfProducts>
  </reaction>
  <reaction id="Vkgdh" reversible="true">
    <listOfReactants>
      <speciesReference species="aKG" stoichiometry="1"/>
      <speciesReference species="NAD" stoichiometry="1"/>
    </listOfReactants>
    <listOfProducts>
      <speciesReference species="SuccCoA" stoichiometry="1"/>
      <speciesReference species="NADH" stoichiometry="1"/>
      <speciesReference species="CO2" stoichiometry="1"/>
    </listOfProducts>
  </reaction>
  <reaction id="Vsdh" reversible="true">
    <listOfReactants>
      <speciesReference species="Succ" stoichiometry="1"/>
      <speciesReference species="FAD" stoichiometry="1"/>
    </listOfReactants>
    <listOfProducts>
      <speciesReference species="Fum" stoichiometry="1"/>
      <speciesReference species="FADH2" stoichiometry="1"/>
    </listOfProducts>
  </reaction>
  <reaction id="Vfum" reversible="true">
```

```
      <listOfReactants>
        <speciesReference species="Fum" stoichiometry="1"/>
      </listOfReactants>
      <listOfProducts>
        <speciesReference species="Mal" stoichiometry="1"/>
      </listOfProducts>
    </reaction>
    <reaction id="Vmdh" reversible="true">
      <listOfReactants>
        <speciesReference species="Mal" stoichiometry="1"/>
        <speciesReference species="NAD" stoichiometry="1"/>
      </listOfReactants>
      <listOfProducts>
        <speciesReference species="OAA" stoichiometry="1"/>
        <speciesReference species="NADH" stoichiometry="1"/>
      </listOfProducts>
    </reaction>
    <reaction id="Vepi" reversible="true">
      <listOfReactants>
        <speciesReference species="Ru5P" stoichiometry="1"/>
      </listOfReactants>
      <listOfProducts>
        <speciesReference species="X5P" stoichiometry="1"/>
      </listOfProducts>
    </reaction>
    <reaction id="Vriso" reversible="true">
      <listOfReactants>
        <speciesReference species="Ru5P" stoichiometry="1"/>
      </listOfReactants>
      <listOfProducts>
        <speciesReference species="Ri5P" stoichiometry="1"/>
      </listOfProducts>
    </reaction>
    <reaction id="Vtk1" reversible="true">
      <listOfReactants>
        <speciesReference species="GAP" stoichiometry="1"/>
        <speciesReference species="S7P" stoichiometry="1"/>
      </listOfReactants>
      <listOfProducts>
        <speciesReference species="Ri5P" stoichiometry="1"/>
        <speciesReference species="X5P" stoichiometry="1"/>
      </listOfProducts>
    </reaction>
    <reaction id="Vtk2" reversible="true">
      <listOfReactants>
        <speciesReference species="F6P" stoichiometry="1"/>
        <speciesReference species="GAP" stoichiometry="1"/>
      </listOfReactants>
      <listOfProducts>
        <speciesReference species="E4P" stoichiometry="1"/>
        <speciesReference species="X5P" stoichiometry="1"/>
      </listOfProducts>
```

```
</reaction>
<reaction id="Vtald" reversible="true">
   <listOfReactants>
      <speciesReference species="GAP" stoichiometry="1"/>
      <speciesReference species="S7P" stoichiometry="1"/>
   </listOfReactants>
   <listOfProducts>
      <speciesReference species="E4P" stoichiometry="1"/>
      <speciesReference species="F6P" stoichiometry="1"/>
   </listOfProducts>
</reaction>
<reaction id="Vpgm" reversible="true">
   <listOfReactants>
      <speciesReference species="G1P" stoichiometry="1"/>
   </listOfReactants>
   <listOfProducts>
      <speciesReference species="G6P" stoichiometry="1"/>
   </listOfProducts>
</reaction>
<reaction id="Vsusy" reversible="true">
   <listOfReactants>
      <speciesReference species="Fru" stoichiometry="1"/>
      <speciesReference species="UDPG" stoichiometry="1"/>
   </listOfReactants>
   <listOfProducts>
      <speciesReference species="Suc" stoichiometry="1"/>
      <speciesReference species="UDP" stoichiometry="1"/>
   </listOfProducts>
</reaction>
<reaction id="Valaat" reversible="true">
   <listOfReactants>
      <speciesReference species="Glu" stoichiometry="1"/>
      <speciesReference species="Pyr" stoichiometry="1"/>
   </listOfReactants>
   <listOfProducts>
      <speciesReference species="aKG" stoichiometry="1"/>
      <speciesReference species="Ala" stoichiometry="1"/>
   </listOfProducts>
</reaction>
<reaction id="Vaspat" reversible="true">
   <listOfReactants>
      <speciesReference species="Glu" stoichiometry="1"/>
      <speciesReference species="OAA" stoichiometry="1"/>
   </listOfReactants>
   <listOfProducts>
      <speciesReference species="aKG" stoichiometry="1"/>
      <speciesReference species="Asp" stoichiometry="1"/>
   </listOfProducts>
</reaction>
<reaction id="Vserat" reversible="true">
   <listOfReactants>
      <speciesReference species="Glu" stoichiometry="1"/>
```

```
      <speciesReference species="PG" stoichiometry="1"/>
      <speciesReference species="NAD" stoichiometry="1"/>
   </listOfReactants>
   <listOfProducts>
      <speciesReference species="aKG" stoichiometry="1"/>
      <speciesReference species="Ser" stoichiometry="1"/>
      <speciesReference species="NADH" stoichiometry="1"/>
   </listOfProducts>
</reaction>
<reaction id="Vgdh" reversible="true">
   <listOfReactants>
      <speciesReference species="aKG" stoichiometry="1"/>
      <speciesReference species="NADH" stoichiometry="1"/>
      <speciesReference species="NH4" stoichiometry="1"/>
   </listOfReactants>
   <listOfProducts>
      <speciesReference species="Glu" stoichiometry="1"/>
      <speciesReference species="NAD" stoichiometry="1"/>
   </listOfProducts>
</reaction>
<reaction id="Vadk" reversible="true">
   <listOfReactants>
      <speciesReference species="AMP" stoichiometry="1"/>
      <speciesReference species="ATP" stoichiometry="1"/>
   </listOfReactants>
   <listOfProducts>
      <speciesReference species="ADP" stoichiometry="2"/>
   </listOfProducts>
</reaction>
<reaction id="Vnar" reversible="true">
   <listOfReactants>
      <speciesReference species="MaloCoA" stoichiometry="3"/>
      <speciesReference species="CoumCoA" stoichiometry="1"/>
   </listOfReactants>
   <listOfProducts>
      <speciesReference species="Naring" stoichiometry="1"/>
      <speciesReference species="CO2" stoichiometry="4"/>
   </listOfProducts>
</reaction>
<reaction id="Vdhq" reversible="true">
   <listOfReactants>
      <speciesReference species="aKG" stoichiometry="1"/>
      <speciesReference species="NADPH" stoichiometry="1"/>
      <speciesReference species="Naring" stoichiometry="1"/>
   </listOfReactants>
   <listOfProducts>
      <speciesReference species="Succ" stoichiometry="1"/>
      <speciesReference species="NADP" stoichiometry="1"/>
      <speciesReference species="Dihydroq" stoichiometry="1"/>
      <speciesReference species="CO2" stoichiometry="1"/>
   </listOfProducts>
</reaction>
```

```
         <reaction id="Vlcc" reversible="true">
            <listOfReactants>
               <speciesReference species="NADPH" stoichiometry="1"/>
               <speciesReference species="Dihydroq" stoichiometry="1"/>
            </listOfReactants>
            <listOfProducts>
               <speciesReference species="NADP" stoichiometry="1"/>
               <speciesReference species="Leucocyan"
stoichiometry="1"/>
            </listOfProducts>
         </reaction>
         <reaction id="Vglc_up" reversible="false">
            <listOfReactants>
               <speciesReference species="Glc_in" stoichiometry="1"/>
            </listOfReactants>
            <listOfProducts>
               <speciesReference species="Glc" stoichiometry="1"/>
            </listOfProducts>
         </reaction>
         <reaction id="Vfru_up" reversible="false">
            <listOfReactants>
               <speciesReference species="Fru_in" stoichiometry="1"/>
            </listOfReactants>
            <listOfProducts>
               <speciesReference species="Fru" stoichiometry="1"/>
            </listOfProducts>
         </reaction>
         <reaction id="Vfk" reversible="false">
            <listOfReactants>
               <speciesReference species="Fru" stoichiometry="1"/>
               <speciesReference species="ATP" stoichiometry="1"/>
            </listOfReactants>
            <listOfProducts>
               <speciesReference species="F6P" stoichiometry="1"/>
               <speciesReference species="ADP" stoichiometry="1"/>
            </listOfProducts>
         </reaction>
         <reaction id="Vgk" reversible="false">
            <listOfReactants>
               <speciesReference species="Glc" stoichiometry="1"/>
               <speciesReference species="ATP" stoichiometry="1"/>
            </listOfReactants>
            <listOfProducts>
               <speciesReference species="G6P" stoichiometry="1"/>
               <speciesReference species="ADP" stoichiometry="1"/>
            </listOfProducts>
         </reaction>
         <reaction id="Vpfk" reversible="false">
            <listOfReactants>
               <speciesReference species="F6P" stoichiometry="1"/>
               <speciesReference species="ATP" stoichiometry="1"/>
            </listOfReactants>
```

```
      <listOfProducts>
         <speciesReference species="F16bP" stoichiometry="1"/>
         <speciesReference species="ADP" stoichiometry="1"/>
      </listOfProducts>
   </reaction>
   <reaction id="Vpfp" reversible="false">
      <listOfReactants>
         <speciesReference species="F6P" stoichiometry="1"/>
         <speciesReference species="PPi" stoichiometry="1"/>
      </listOfReactants>
      <listOfProducts>
         <speciesReference species="F16bP" stoichiometry="1"/>
      </listOfProducts>
   </reaction>
   <reaction id="Vfbp" reversible="false">
      <listOfReactants>
         <speciesReference species="F16bP" stoichiometry="1"/>
      </listOfReactants>
      <listOfProducts>
         <speciesReference species="F6P" stoichiometry="1"/>
      </listOfProducts>
   </reaction>
   <reaction id="Vpk" reversible="false">
      <listOfReactants>
         <speciesReference species="PEP" stoichiometry="1"/>
         <speciesReference species="ADP" stoichiometry="1"/>
      </listOfReactants>
      <listOfProducts>
         <speciesReference species="Pyr" stoichiometry="1"/>
         <speciesReference species="ATP" stoichiometry="1"/>
      </listOfProducts>
   </reaction>
   <reaction id="Vpepc" reversible="false">
      <listOfReactants>
         <speciesReference species="PEP" stoichiometry="1"/>
         <speciesReference species="CO2" stoichiometry="1"/>
      </listOfReactants>
      <listOfProducts>
         <speciesReference species="OAA" stoichiometry="1"/>
      </listOfProducts>
   </reaction>
   <reaction id="Vpdh" reversible="false">
      <listOfReactants>
         <speciesReference species="Pyr" stoichiometry="1"/>
         <speciesReference species="NAD" stoichiometry="1"/>
      </listOfReactants>
      <listOfProducts>
         <speciesReference species="AcCoA" stoichiometry="1"/>
         <speciesReference species="NADH" stoichiometry="1"/>
         <speciesReference species="CO2" stoichiometry="1"/>
      </listOfProducts>
   </reaction>
```

```
<reaction id="Vcs" reversible="false">
   <listOfReactants>
      <speciesReference species="AcCoA" stoichiometry="1"/>
      <speciesReference species="OAA" stoichiometry="1"/>
   </listOfReactants>
   <listOfProducts>
      <speciesReference species="Cit" stoichiometry="1"/>
   </listOfProducts>
</reaction>
<reaction id="Vme" reversible="false">
   <listOfReactants>
      <speciesReference species="Mal" stoichiometry="1"/>
      <speciesReference species="NAD" stoichiometry="1"/>
   </listOfReactants>
   <listOfProducts>
      <speciesReference species="Pyr" stoichiometry="1"/>
      <speciesReference species="NADH" stoichiometry="1"/>
      <speciesReference species="CO2" stoichiometry="1"/>
   </listOfProducts>
</reaction>
<reaction id="Vscol" reversible="false">
   <listOfReactants>
      <speciesReference species="SuccCoA" stoichiometry="1"/>
      <speciesReference species="ADP" stoichiometry="1"/>
   </listOfReactants>
   <listOfProducts>
      <speciesReference species="Succ" stoichiometry="1"/>
      <speciesReference species="ATP" stoichiometry="1"/>
   </listOfProducts>
</reaction>
<reaction id="Vg6pdh" reversible="false">
   <listOfReactants>
      <speciesReference species="G6P" stoichiometry="1"/>
      <speciesReference species="NADP" stoichiometry="2"/>
   </listOfReactants>
   <listOfProducts>
      <speciesReference species="Ru5P" stoichiometry="1"/>
      <speciesReference species="NADPH" stoichiometry="2"/>
      <speciesReference species="CO2" stoichiometry="1"/>
   </listOfProducts>
</reaction>
<reaction id="Vagpase" reversible="false">
   <listOfReactants>
      <speciesReference species="G1P" stoichiometry="1"/>
      <speciesReference species="ATP" stoichiometry="1"/>
   </listOfReactants>
   <listOfProducts>
      <speciesReference species="ADPG" stoichiometry="1"/>
      <speciesReference species="PPi" stoichiometry="1"/>
   </listOfProducts>
</reaction>
<reaction id="Vugpase" reversible="false">
```

```xml
      <listOfReactants>
        <speciesReference species="G1P" stoichiometry="1"/>
        <speciesReference species="UTP" stoichiometry="1"/>
      </listOfReactants>
      <listOfProducts>
        <speciesReference species="UDPG" stoichiometry="1"/>
        <speciesReference species="PPi" stoichiometry="1"/>
      </listOfProducts>
    </reaction>
    <reaction id="Vinv" reversible="false">
      <listOfReactants>
        <speciesReference species="Suc" stoichiometry="1"/>
      </listOfReactants>
      <listOfProducts>
        <speciesReference species="Fru" stoichiometry="1"/>
        <speciesReference species="Glc" stoichiometry="1"/>
      </listOfProducts>
    </reaction>
    <reaction id="Vsps" reversible="false">
      <listOfReactants>
        <speciesReference species="F6P" stoichiometry="1"/>
        <speciesReference species="UDPG" stoichiometry="1"/>
      </listOfReactants>
      <listOfProducts>
        <speciesReference species="SucP" stoichiometry="1"/>
        <speciesReference species="UDP" stoichiometry="1"/>
      </listOfProducts>
    </reaction>
    <reaction id="Vspase" reversible="false">
      <listOfReactants>
        <speciesReference species="SucP" stoichiometry="1"/>
      </listOfReactants>
      <listOfProducts>
        <speciesReference species="Suc" stoichiometry="1"/>
      </listOfProducts>
    </reaction>
    <reaction id="Vno3_up" reversible="false">
      <listOfReactants>
        <speciesReference species="NO3_in" stoichiometry="1"/>
      </listOfReactants>
      <listOfProducts>
        <speciesReference species="NO3" stoichiometry="1"/>
      </listOfProducts>
    </reaction>
    <reaction id="Vgs" reversible="false">
      <listOfReactants>
        <speciesReference species="Glu" stoichiometry="1"/>
        <speciesReference species="ATP" stoichiometry="1"/>
        <speciesReference species="NH4" stoichiometry="1"/>
      </listOfReactants>
      <listOfProducts>
        <speciesReference species="Gln" stoichiometry="1"/>
```

```
      <speciesReference species="ADP" stoichiometry="1"/>
    </listOfProducts>
  </reaction>
  <reaction id="Vgogat" reversible="false">
    <listOfReactants>
      <speciesReference species="aKG" stoichiometry="1"/>
      <speciesReference species="Gln" stoichiometry="1"/>
      <speciesReference species="NADPH" stoichiometry="1"/>
    </listOfReactants>
    <listOfProducts>
      <speciesReference species="Glu" stoichiometry="2"/>
      <speciesReference species="NADP" stoichiometry="1"/>
    </listOfProducts>
  </reaction>
  <reaction id="Vnr" reversible="false">
    <listOfReactants>
      <speciesReference species="NADH" stoichiometry="1"/>
      <speciesReference species="NO3" stoichiometry="1"/>
    </listOfReactants>
    <listOfProducts>
      <speciesReference species="NAD" stoichiometry="1"/>
      <speciesReference species="NH4" stoichiometry="1"/>
    </listOfProducts>
  </reaction>
  <reaction id="Vmacl" reversible="false">
    <listOfReactants>
      <speciesReference species="AcCoA" stoichiometry="1"/>
      <speciesReference species="CO2" stoichiometry="1"/>
    </listOfReactants>
    <listOfProducts>
      <speciesReference species="MaloCoA" stoichiometry="1"/>
    </listOfProducts>
  </reaction>
  <reaction id="Vshik" reversible="false">
    <listOfReactants>
      <speciesReference species="E4P" stoichiometry="1"/>
      <speciesReference species="PEP" stoichiometry="1"/>
      <speciesReference species="NADPH" stoichiometry="1"/>
      <speciesReference species="ATP" stoichiometry="1"/>
    </listOfReactants>
    <listOfProducts>
      <speciesReference species="NADP" stoichiometry="1"/>
      <speciesReference species="ADP" stoichiometry="1"/>
      <speciesReference species="Shik" stoichiometry="1"/>
    </listOfProducts>
  </reaction>
  <reaction id="Vphe" reversible="false">
    <listOfReactants>
      <speciesReference species="Glu" stoichiometry="1"/>
      <speciesReference species="Shik" stoichiometry="1"/>
    </listOfReactants>
    <listOfProducts>
```

```
      <speciesReference species="aKG" stoichiometry="1"/>
      <speciesReference species="Phe" stoichiometry="1"/>
      <speciesReference species="CO2" stoichiometry="1"/>
    </listOfProducts>
  </reaction>
  <reaction id="Vpal" reversible="false">
    <listOfReactants>
      <speciesReference species="Phe" stoichiometry="1"/>
    </listOfReactants>
    <listOfProducts>
      <speciesReference species="NH4" stoichiometry="1"/>
      <speciesReference species="Cinn" stoichiometry="1"/>
    </listOfProducts>
  </reaction>
  <reaction id="Vcoum" reversible="false">
    <listOfReactants>
      <speciesReference species="NADPH" stoichiometry="1"/>
      <speciesReference species="ATP" stoichiometry="1"/>
      <speciesReference species="Cinn" stoichiometry="1"/>
    </listOfReactants>
    <listOfProducts>
      <speciesReference species="NADP" stoichiometry="1"/>
      <speciesReference species="AMP" stoichiometry="1"/>
      <speciesReference species="PPi" stoichiometry="1"/>
      <speciesReference species="CoumCoA" stoichiometry="1"/>
    </listOfProducts>
  </reaction>
  <reaction id="Vnrj1" reversible="false">
    <listOfReactants>
      <speciesReference species="NADH" stoichiometry="1"/>
      <speciesReference species="ADP" stoichiometry="2"/>
    </listOfReactants>
    <listOfProducts>
      <speciesReference species="NAD" stoichiometry="1"/>
      <speciesReference species="ATP" stoichiometry="2"/>
    </listOfProducts>
  </reaction>
  <reaction id="Vnrj2" reversible="false">
    <listOfReactants>
      <speciesReference species="ADP" stoichiometry="1.5"/>
      <speciesReference species="FADH2" stoichiometry="1"/>
    </listOfReactants>
    <listOfProducts>
      <speciesReference species="ATP" stoichiometry="1.5"/>
      <speciesReference species="FAD" stoichiometry="1"/>
    </listOfProducts>
  </reaction>
  <reaction id="Vnrj3" reversible="false">
    <listOfReactants>
      <speciesReference species="ATP" stoichiometry="1"/>
      <speciesReference species="UDP" stoichiometry="1"/>
    </listOfReactants>
```

```
            &lt;listOfProducts&gt;
               &lt;speciesReference species="ADP" stoichiometry="1"/&gt;
               &lt;speciesReference species="UTP" stoichiometry="1"/&gt;
            &lt;/listOfProducts&gt;
         &lt;/reaction&gt;
         &lt;reaction id="Vnga_ATP" reversible="false"&gt;
            &lt;listOfReactants&gt;
               &lt;speciesReference species="ATP" stoichiometry="1"/&gt;
            &lt;/listOfReactants&gt;
            &lt;listOfProducts&gt;
               &lt;speciesReference species="ADP" stoichiometry="1"/&gt;
            &lt;/listOfProducts&gt;
         &lt;/reaction&gt;
         &lt;reaction id="Vppi" reversible="false"&gt;
            &lt;listOfReactants&gt;
               &lt;speciesReference species="PPi" stoichiometry="1"/&gt;
            &lt;/listOfReactants&gt;
         &lt;/reaction&gt;
         &lt;reaction id="Vanthoc" reversible="false"&gt;
            &lt;listOfReactants&gt;
               &lt;speciesReference species="aKG" stoichiometry="1"/&gt;
               &lt;speciesReference species="UDPG" stoichiometry="1"/&gt;
               <speciesReference species="Leucocyan"
stoichiometry="1"/>
            &lt;/listOfReactants&gt;
            &lt;listOfProducts&gt;
               &lt;speciesReference species="Succ" stoichiometry="1"/&gt;
               &lt;speciesReference species="UDP" stoichiometry="1"/&gt;
               &lt;speciesReference species="Anthoc" stoichiometry="1"/&gt;
               &lt;speciesReference species="CO2" stoichiometry="1"/&gt;
            &lt;/listOfProducts&gt;
         &lt;/reaction&gt;
         &lt;reaction id="Vflav" reversible="false"&gt;
            &lt;listOfReactants&gt;
               &lt;speciesReference species="aKG" stoichiometry="1"/&gt;
               &lt;speciesReference species="UDPG" stoichiometry="1"/&gt;
               &lt;speciesReference species="Dihydroq" stoichiometry="1"/&gt;
            &lt;/listOfReactants&gt;
            &lt;listOfProducts&gt;
               &lt;speciesReference species="Succ" stoichiometry="1"/&gt;
               &lt;speciesReference species="UDP" stoichiometry="1"/&gt;
               &lt;speciesReference species="Flav" stoichiometry="1"/&gt;
               &lt;speciesReference species="CO2" stoichiometry="1"/&gt;
            &lt;/listOfProducts&gt;
         &lt;/reaction&gt;
         &lt;reaction id="Vstilb" reversible="false"&gt;
            &lt;listOfReactants&gt;
               &lt;speciesReference species="MaloCoA" stoichiometry="3"/&gt;
               &lt;speciesReference species="CoumCoA" stoichiometry="1"/&gt;
            &lt;/listOfReactants&gt;
            &lt;listOfProducts&gt;
               &lt;speciesReference species="Stilb" stoichiometry="1"/&gt;
```

```
          <speciesReference species="CO2" stoichiometry="4"/>
        </listOfProducts>
      </reaction>
      <reaction id="Vtanins" reversible="false">
        <listOfReactants>
          <speciesReference species="NADPH" stoichiometry="1"/>
          <speciesReference species="Leucocyan"
stoichiometry="1"/>
        </listOfReactants>
        <listOfProducts>
          <speciesReference species="NADP" stoichiometry="1"/>
          <speciesReference species="Tanins" stoichiometry="1"/>
        </listOfProducts>
      </reaction>
      <reaction id="Vcw" reversible="false">
        <listOfReactants>
          <speciesReference species="UDPG" stoichiometry="1"/>
        </listOfReactants>
        <listOfProducts>
          <speciesReference species="UDP" stoichiometry="1"/>
          <speciesReference species="CW" stoichiometry="1"/>
        </listOfProducts>
      </reaction>
      <reaction id="Vac_glu" reversible="false">
        <listOfReactants>
          <speciesReference species="Glu" stoichiometry="1"/>
        </listOfReactants>
        <listOfProducts>
          <speciesReference species="Glu_ac" stoichiometry="1"/>
        </listOfProducts>
      </reaction>
      <reaction id="Vac_asp" reversible="false">
        <listOfReactants>
          <speciesReference species="Asp" stoichiometry="1"/>
        </listOfReactants>
        <listOfProducts>
          <speciesReference species="Asp_ac" stoichiometry="1"/>
        </listOfProducts>
      </reaction>
      <reaction id="Vac_ala" reversible="false">
        <listOfReactants>
          <speciesReference species="Ala" stoichiometry="1"/>
        </listOfReactants>
        <listOfProducts>
          <speciesReference species="Ala_ac" stoichiometry="1"/>
        </listOfProducts>
      </reaction>
      <reaction id="Vac_phe" reversible="false">
        <listOfReactants>
          <speciesReference species="Phe" stoichiometry="1"/>
        </listOfReactants>
        <listOfProducts>
```

```
      <speciesReference species="Phe_ac" stoichiometry="1"/>
    </listOfProducts>
  </reaction>
  <reaction id="Vprotein" reversible="false">
    <listOfReactants>
      <speciesReference species="Ala" stoichiometry="0.33"/>
      <speciesReference species="Asp" stoichiometry="0.23"/>
      <speciesReference species="Glu" stoichiometry="0.26"/>
      <speciesReference species="Ser" stoichiometry="0.09"/>
      <speciesReference species="NADPH" stoichiometry="0.32"/>
      <speciesReference species="ATP" stoichiometry="4.3"/>
      <speciesReference species="Phe" stoichiometry="0.09"/>
    </listOfReactants>
    <listOfProducts>
      <speciesReference species="NADP" stoichiometry="0.32"/>
      <speciesReference species="ADP" stoichiometry="4.3"/>
      <speciesReference species="PROTEIN" stoichiometry="1"/>
    </listOfProducts>
  </reaction>
  <reaction id="Vac_mal" reversible="false">
    <listOfReactants>
      <speciesReference species="Mal" stoichiometry="1"/>
    </listOfReactants>
    <listOfProducts>
      <speciesReference species="Mal_ac" stoichiometry="1"/>
    </listOfProducts>
  </reaction>
  <reaction id="Vac_glc" reversible="false">
    <listOfReactants>
      <speciesReference species="Glc" stoichiometry="1"/>
    </listOfReactants>
    <listOfProducts>
      <speciesReference species="Glc_ac" stoichiometry="1"/>
    </listOfProducts>
  </reaction>
  <reaction id="Vac_fru" reversible="false">
    <listOfReactants>
      <speciesReference species="Fru" stoichiometry="1"/>
    </listOfReactants>
    <listOfProducts>
      <speciesReference species="Fru_ac" stoichiometry="1"/>
    </listOfProducts>
  </reaction>
  <reaction id="Vac_suc" reversible="false">
    <listOfReactants>
      <speciesReference species="Suc" stoichiometry="1"/>
    </listOfReactants>
    <listOfProducts>
      <speciesReference species="Suc_ac" stoichiometry="1"/>
    </listOfProducts>
  </reaction>
  <reaction id="Vss" reversible="false">
```

```
          <listOfReactants>
            <speciesReference species="ADPG" stoichiometry="1"/>
          </listOfReactants>
          <listOfProducts>
            <speciesReference species="ADP" stoichiometry="1"/>
            <speciesReference species="STARCH" stoichiometry="1"/>
          </listOfProducts>
        </reaction>
        <reaction id="Vnucleo" reversible="false">
          <listOfReactants>
            <speciesReference species="Asp" stoichiometry="2.1"/>
            <speciesReference species="Gln" stoichiometry="6.3"/>
            <speciesReference species="Ri5P" stoichiometry="3"/>
            <speciesReference species="ATP" stoichiometry="21.9"/>
            <speciesReference species="CO2" stoichiometry="3"/>
          </listOfReactants>
          <listOfProducts>
            <speciesReference species="Fum" stoichiometry="2.1"/>
            <speciesReference species="Glu" stoichiometry="6.3"/>
            <speciesReference species="AMP" stoichiometry="4.5"/>
            <speciesReference species="ADP" stoichiometry="17.4"/>
            <speciesReference species="PPi" stoichiometry="3"/>
            <speciesReference species="ADNARN" stoichiometry="1"/>
          </listOfProducts>
        </reaction>
      </listOfReactions>
    </model>
  </sbml>
```
